# Supplementary material for: Grade repetition and bullying victimization in adolescents: A global cross-sectional study of the Program for International Student Assessment (PISA) data from 2018
Source: PLoS Med. 2021 Nov 11;18(11):e1003846. doi: 10.1371/journal.pmed.1003846 (PMC8584722; doi:10.1371/journal.pmed.1003846)
Supplement: S1 Table — (DOCX) [file pmed.1003846.s001.docx]

S1 Table. Sample size in this study, by country/economy

| Country/economy | n | Country/economy | n |
| --- | --- | --- | --- |
| Albania | 6,084 | Lithuania | 5,595 |
| Baku (Azerbaijan) | 3,828 | Luxembourg | 4,616 |
| Argentina | 7,903 | Macao, China | 3,761 |
| Australia | 10,692 | Malta | 2,824 |
| Austria | 5,387 | Mexico | 3,918 |
| Belgium | 7,294 | Moldova | 4,958 |
| Bosnia and Herzegovin | 5,556 | Montenegro | 5,685 |
| Brazil | 6,711 | Morocco | 2,734 |
| Brunei Darussalam | 4,818 | Netherlands | 3,626 |
| Bulgaria | 3,955 | New Zealand | 5,064 |
| Belarus | 5,452 | Panama | 1,966 |
| Canada | 19,041 | Peru | 2,199 |
| Chile | 5,056 | Philippines | 6,341 |
| Taiwan, China | 7,078 | Poland | 5,092 |
| Colombia | 5,304 | Portugal | 4,901 |
| Costa Rica | 6,337 | Qatar | 11,755 |
| Croatia | 5,581 | Romania | 4,560 |
| Czech Republic | 6,236 | Russian Federation | 6,586 |
| Denmark | 6,064 | Saudi Arabia | 5,311 |
| Dominican Republic | 1,663 | Serbia | 4,976 |
| Estonia | 4,847 | Singapore | 6,419 |
| Finland | 5,069 | Slovak Republic | 4,951 |
| France | 4,817 | Vietnam | 5,283 |
| Georgia | 4,600 | Slovenia | 5,041 |
| Germany | 2,386 | Spain | 27,321 |
| Greece | 5,613 | Sweden | 4,824 |
| Hong Kong, China | 5,598 | Switzerland | 3,630 |
| Hungary | 4,337 | Thailand | 8,367 |
| Iceland | 2,461 | United Arab Emirates | 17,337 |
| Indonesia | 11,771 | Turkey | 6,670 |
| Ireland | 4,554 | Ukraine | 5,079 |
| Italy | 8,858 | United Kingdom | 12,275 |
| Kosovo | 4,574 | United States | 4,558 |
| Kazakhstan | 16,096 | Uruguay | 3,064 |
| Jordan | 8,076 | B-S-J-Z^*^ (China) | 11,956 |
| Korea | 6,595 | Moscow Region (RUS) | 1,776 |
| Latvia | 4,584 | Tatarstan (RUS) | 5,251 |

^*^ B-S-J-Z refers to the four PISA participating China provinces: Beijing, Shanghai, Jiangsu, and Zhejiang.
