# Supplementary material for: Grade repetition and bullying victimization in adolescents: A global cross-sectional study of the Program for International Student Assessment (PISA) data from 2018
Source: PLoS Med. 2021 Nov 11;18(11):e1003846. doi: 10.1371/journal.pmed.1003846 (PMC8584722; doi:10.1371/journal.pmed.1003846)
Supplement: S3 Table — (DOCX) [file pmed.1003846.s003.docx]

S3 Table. Measures related questions in PISA 2018

| Measures | PISA 2018 questions |
| --- | --- |
| Grade repetition | Have you ever repeated a <grade>? (Please select one response in each row)  Response: □No, never; □Yes, once; □Yes, twice or more |
|  | At <ISCED 1> |
|  | At <ISCED 2> |
|  | At <ISCED 3> |
| Bullying victimization | During the past 12 months, how often have you had the following experiences in school? (Some experiences can also happen in social media)  Response: □Never or almost never; □A few times a year;  □A few times a month; □Once a week or more |
|  | 1) Other students left me out of things on purpose |
|  | 2) Other students made fun of me |
|  | 3) I was threatened by other students |
|  | 4) Other students took away or destroyed things that belong to me |
|  | 5) I got hit or pushed around by other students |
|  | 6) Other students spread nasty rumours about me |
| Sex | Are you female or male?  Response: □Female; □Male |
| Age | On what date were you born?  (Please select the day, month, and year from the drop-down menus to answer the question.) |
| Migrant status | In what country were you and your parents born?  You, Mother, Father |
| School type | Is your school a public or a private school?  (Please select one response.)  □A public school (This is a school managed directly or indirectly by  a public education authority, government agency, or governing  board appointed by government or elected by public franchise.)  □A private school (This is a school managed directly or indirectly  by a non-government organisation; e.g. a church, trade union,  business, or other private institution.) |
| ESCS: parental highest occupational status | The following two questions concern your mother’s job:  (If she is not working now, please tell us her last main job.) |
|  | What’s your mother’s main job?  (e.g. school teacher, kitchen-hand, sales manager)  Please type in the job title. _________ |
|  | What does your mother do in her/his main job?  (e.g. teaches high school students, helps the cook prepare meals in a  restaurant, manages a sales team) Please use a sentence to describe the kind of work she does or did  in that job.__________ |
|  | The following two questions concern your father’s job:  (If he is not working now, please tell us his last main job.) |
|  | What’s your father’s main job?  (e.g. school teacher, kitchen-hand, sales manager)  Please type in the job title. _________ |
|  | What does your father do in her/his main job?  (e.g. teaches high school students, helps the cook prepare meals in a  restaurant, manages a sales team)  Please use a sentence to describe the kind of work she does or did  in that job.__________ |
| ESCS: parental educational attainment | What is the <highest level of schooling> completed by your mother? |
|  | Does your mother have any of the following qualifications? |
|  | What is the <highest level of schooling> completed by your father? |
|  | Does your father have any of the following qualifications? |
| ESCS: home possessions/ household items | Which of the following are in your home?  (Please select one response in each row.)  Response: □Yes; □No |
|  | A desk to study at |
|  | A room of your own |
|  | A quiet place to study |
|  | A computer you can use for school work |
|  | Educational software |
|  | A link to the Internet |
|  | Classic literature |
|  | Books of poetry |
|  | Works of art (e.g. paintings) |
|  | Books to help with your school work |
|  | <Technical reference books> |
|  | A dictionary |
|  | Books on art, music, or design |
|  | <Country-specific wealth item 1> |
|  | <Country-specific wealth item 2> |
|  | <Country-specific wealth item 3> |
| ESCS: home possessions/amount of possessions | How many of these are there at your home?  (Please select one response in each row.)  Response: □None; □One ; □Two; □Three or more |
|  | Televisions |
|  | Cars |
|  | Rooms with a bath or shower |
|  | <Cell phones> with Internet access (e.g. smartphones) |
|  | Computers (desktop computer, portable laptop, or notebook) |
|  | <Tablet computers> |
|  | E-book readers |
|  | Musical instruments (e.g. guitar, piano) |
| ESCS: home possessions/number of books | How many books are there in your home?  There are usually about 40 books per metre of shelving. Do not  include magazines, newspapers, or your schoolbooks.  (Please select one response.) |
|  | 0-10 books |
|  | 11-25 books |
|  | 26-100 books |
|  | 101-200 books |
|  | 201-500 books |
|  | More than 500 books |
| Parental emotional support | Thinking about <this academic year>: to what extent do you  agree or disagree with the following statements?  (Please select one response in each row.)  Response: □Strongly disagree; □Disagree; □Agree ; □Strongly  agree |
|  | My parents support my educational efforts and achievements. |
|  | My parents support me when I am facing difficulties at school. |
|  | My parents encourage me to be confident. |

Abbreviation: ISCED, International Standard Classification of Education; ISCED 1, the primary education level; ISCED 2, the lower secondary education level; ISCED 3, the upper secondary education level; ESCS: Economic, Social and Culture Status
