# Supplementary material for: Grade repetition and bullying victimization in adolescents: A global cross-sectional study of the Program for International Student Assessment (PISA) data from 2018
Source: PLoS Med. 2021 Nov 11;18(11):e1003846. doi: 10.1371/journal.pmed.1003846 (PMC8584722; doi:10.1371/journal.pmed.1003846)
Supplement: S4 Table — (DOCX) [file pmed.1003846.s004.docx]

S4 Table. Country-specific description of age

| Country/economy | Mean (SD)[Range] | Country/economy | Mean (SD)[Range] |
| --- | --- | --- | --- |
| Albania | 15.78(0.30) [15.33-16.33] | Lithuania | 15.81(0.29) [15.33-16.33] |
| Baku (Azerbaijan) | 15.83(0.29) [15.33-16.33] | Luxembourg | 15.83(0.29) [15.33-16.33] |
| Argentina | 15.71(0.28) [15.25-16.17] | Macao (China) | 15.83(0.29) [15.33-16.33] |
| Australia | 15.79(0.29) [15.25-16.33] | Malta | 15.69(0.29) [15.25-16.17] |
| Austria | 15.80(0.29) [15.33-16.33] | Mexico | 15.84(0.28) [15.33-16.33] |
| Belgium | 15.85(0.29) [15.33-16.33] | Moldova | 15.70(0.28) [15.25-16.17] |
| Bosnia and Herzegovin | 15.79(0.28) [15.33-16.33] | Montenegro | 15.78(0.29) [15.25-16.25] |
| Brazil | 15.89(0.28) [15.42-16.33] | Morocco | 15.86(0.29) [15.33-16.33] |
| Brunei Darussalam | 15.79(0.29) [15.33-16.33] | Netherlands | 15.75(0.29) [15.25-16.25] |
| Bulgaria | 15.79(0.29) [15.33-16.33] | New Zealand | 15.78(0.29) [15.25-16.33] |
| Belarus | 15.79(0.28) [15.33-16.33] | Panama | 15.80(0.28) [15.33-16.33] |
| Canada | 15.85(0.29) [15.33-16.33] | Peru | 15.85(0.30) [15.33-16.33] |
| Chile | 15.80(0.28) [15.33-16.33] | Philippines | 15.69(0.29) [15.25-16.25] |
| Taiwan (China) | 15.77(0.30) [15.25-16.33] | Poland | 15.73(0.28) [15.25-16.25] |
| Colombia | 15.84(0.28) [15.33-16.33] | Portugal | 15.75(0.29) [15.25-16.25] |
| Costa Rica | 15.72(0.28) [15.25-16.25] | Qatar | 15.73(0.29) [15.25-16.25] |
| Croatia | 15.74(0.29) [15.25-16.25] | Romania | 15.71(0.28) [15.25-16.25] |
| Czech Republic | 15.79(0.28) [15.25-16.33] | Russian Federation | 15.81(0.29) [15.33-16.33] |
| Denmark | 15.77(0.29) [15.25-16.25] | Saudi Arabia | 15.78(0.28) [15.33-16.25] |
| Dominican Republic | 15.75(0.30) [15.25-16.25] | Serbia | 15.86(0.29) [15.25-16.33] |
| Estonia | 15.82(0.29) [15.33-16.33] | Singapore | 15.78(0.29) [15.33-16.33] |
| Finland | 15.71(0.29) [15.25-16.25] | Slovak Republic | 15.79(0.29) [15.33-16.25] |
| France | 15.87(0.29) [15.42-16.33] | Vietnam | 15.78(0.29) [15.33-16.25] |
| Georgia | 15.84(0.29) [15.33-16.33] | Slovenia | 15.73(0.28) [15.25-16.25] |
| Germany | 15.84(0.29) [15.33-16.33] | Spain | 15.83(0.29) [15.33-16.33] |
| Greece | 15.70(0.29) [15.25-16.17] | Sweden | 15.72(0.28) [15.25-16.17] |
| Hong Kong (China) | 15.74(0.29) [15.25-16.25] | Switzerland | 15.81(0.28) [15.33-16.33] |
| Hungary | 15.78(0.29) [15.25-16.25] | Thailand | 15.71(0.29) [15.25-16.17] |
| Iceland | 15.75(0.29) [15.08-16.25] | United Arab Emirates | 15.82(0.29) [15.33-16.33] |
| Indonesia | 15.85(0.28) [15.33-16.33] | Turkey | 15.82(0.29) [15.33-16.33] |
| Ireland | 15.71(0.29) [15.25-16.25] | Ukraine | 15.80(0.29) [15.33-16.33] |
| Italy | 15.77(0.29) [15.25-16.33] | United Kingdom | 15.76(0.28) [15.25-16.33] |
| Kosovo | 15.78(0.28) [15.33-16.25] | United States | 15.85(0.29) [15.33-16.33] |
| Kazakhstan | 15.80(0.29) [15.33-16.33] | Uruguay | 15.78(0.29) [15.33-16.33] |
| Jordan | 15.86(0.29) [15.42-16.33] | B-S-J-Z ^*^(China) | 15.75(0.30) [15.33-16.25] |
| Korea | 15.72(0.30) [15.25-16.25] | Moscow Region (RUS) | 15.79(0.28) [15.33-16.25] |
| Latvia | 15.78(0.29) [15.25-16.33] | Tatarstan (RUS) | 15.79(0.28) [15.33-16.25] |

^*^ B-S-J-Z refers to the four PISA participating China provinces: Beijing, Shanghai, Jiangsu, and Zhejiang.
