# Supplementary material for: Grade repetition and bullying victimization in adolescents: A global cross-sectional study of the Program for International Student Assessment (PISA) data from 2018
Source: PLoS Med. 2021 Nov 11;18(11):e1003846. doi: 10.1371/journal.pmed.1003846 (PMC8584722; doi:10.1371/journal.pmed.1003846)
Supplement: S5 Table — (DOCX) [file pmed.1003846.s005.docx]

S5 Table. Associations of grade repetition and sex interaction with bullying victimization

| **Outcome** | Strength of association | |
| --- | --- | --- |
| **Types of Bullying victimization** | Crude OR (95%CI, p value) | Adjusted^*^ OR (95%CI, p value) |
| Other students left me out of things on purpose. |  |  |
| Grade repetition (Yes) | 1.47(1.32-1.64, <0.001) | 1.32(1.17-1.48, <0.001) |
| Sex (Male) | 1.19(1.13-1.25, <0.001) | 1.23(1.16-1.31, <0.001) |
| Grade repetition * Sex | 0.96(0.85-1.08, 0.485) | 0.94(0.83-1.07, 0.361) |
| Other students made fun of me. |  |  |
| Grade repetition (Yes) | 1.56(1.42-1.71, <0.001) | 1.38(1.24-1.53, <0.001) |
| Sex (Male) | 1.37(1.30-1.45, <0.001) | 1.41(1.32-1.50, <0.001) |
| Grade repetition * Sex | 0.84(0.75-0.93, 0.001) | 0.80(0.72-0.90, <0.001) |
| I was threatened by other students. |  |  |
| Grade repetition (Yes) | 2.48(2.16-2.86, <0.001) | 2.01(1.72-2.35, <0.001) |
| Sex (Male) | 1.84(1.69-1.99, <0.001) | 1.95(1.78-2.12, <0.001) |
| Grade repetition * Sex | 0.79(0.67-0.93, 0.005) | 0.77(0.64-0.92, 0.005) |
| Other students took away or destroyed things that belonged to me. |  |  |
| Grade repetition (Yes) | 2.31(2.05-2.60, <0.001) | 1.98(1.73-2.27, <0.001) |
| Sex (Male) | 1.67(1.56-1.79, <0.001) | 1.75(1.62-1.88, <0.001) |
| Grade repetition * Sex | 0.79(0.69-0.92, 0.002) | 0.73(0.62-0.85, <0.001) |
| I got hit or pushed around by other students. |  |  |
| Grade repetition (Yes) | 2.41(2.11-2.76, <0.001) | 1.97(1.69-2.29, <0.001) |
| Sex (Male) | 1.96(1.81-2.13, <0.001) | 2.04(1.88-2.22, <0.001) |
| Grade repetition * Sex | 0.83(0.70-0.97, 0.024) | 0.80(0.67-0.96, 0.016) |
| Other students spread nasty rumours about me. |  |  |
| Grade repetition (Yes) | 1.94(1.76-2.15, <0.001) | 1.64(1.46-1.84, <0.001) |
| Sex (Male) | 1.25(1.18-1.34, <0.001) | 1.27(1.19-1.36, <0.001) |
| Grade repetition * Sex | 1.02(0.90-1.16, 0.753) | 1.01(0.88-1.16, 0.913) |
| Any type of victimization |  |  |
| Grade repetition (Yes) | 1.69(1.55-1.84, <0.001) | 1.51(1.36-1.68, <0.001) |
| Sex (Male) | 1.34(1.28-1.41, <0.001) | 1.38(1.30-1.46, <0.001) |
| Grade repetition * Sex | 0.94(0.85-1.03, 0.188) | 0.90(0.80-1.00, 0.056) |

^*^ All models adjusted country, sex, age group, migrant status, school type, economic, social and culture status, and parental emotional support.
