# Supplementary material for: Grade repetition and bullying victimization in adolescents: A global cross-sectional study of the Program for International Student Assessment (PISA) data from 2018
Source: PLoS Med. 2021 Nov 11;18(11):e1003846. doi: 10.1371/journal.pmed.1003846 (PMC8584722; doi:10.1371/journal.pmed.1003846)
Supplement: S6 Table — (DOCX) [file pmed.1003846.s006.docx]

S6 Table. Country-specific prevalence of grade repetition and any type of bullying victimization, weighted %

| Country/economy | Repetition | Victim | Country/economy | Repetition | Victim |
| --- | --- | --- | --- | --- | --- |
| Albania | 3.07 | 25.41 | Lithuania | 2.12 | 22.60 |
| Baku (Azerbaijan) | 2.53 | 35.76 | Luxembourg | 30.44 | 20.55 |
| Argentina | 26.39 | 32.19 | Macao (China) | 29.98 | 27.03 |
| Australia | 5.51 | 29.51 | Malta | 4.83 | 31.82 |
| Austria | 13.05 | 23.16 | Mexico | 9.47 | 22.95 |
| Belgium | 29.79 | 18.59 | Moldova | 2.36 | 23.94 |
| Bosnia and Herzegovin | 4.22 | 33.76 | Montenegro | 1.41 | 25.03 |
| Brazil | 1.69 | 25.24 | Morocco | 42.53 | 43.73 |
| Brunei Darussalam | 1.28 | 18.51 | Netherlands | 17.06 | 12.16 |
| Bulgaria | 30.21 | 28.95 | New Zealand | 5.28 | 31.55 |
| Belarus | 11.87 | 50.13 | Panama | 24.75 | 33.25 |
| Canada | 4.81 | 25.23 | Peru | 12.83 | 22.41 |
| Chile | 20.75 | 23.82 | Philippines | 19.42 | 64.81 |
| Taiwan (China) | 0.81 | 13.28 | Poland | 3.07 | 26.29 |
| Colombia | 38.36 | 32.30 | Portugal | 25.43 | 13.69 |
| Costa Rica | 27.14 | 24.22 | Qatar | 16.36 | 33.04 |
| Croatia | 1.53 | 18.17 | Romania | 4.30 | 33.81 |
| Czech Republic | 4.22 | 29.70 | Russian Federation | 1.65 | 36.57 |
| Denmark | 2.94 | 21.38 | Saudi Arabia | 11.29 | 29.68 |
| Dominican Republic | 27.58 | 43.83 | Serbia | 1.38 | 25.50 |
| Estonia | 2.81 | 25.41 | Singapore | 4.71 | 25.98 |
| Finland | 3.06 | 17.67 | Slovak Republic | 5.12 | 28.25 |
| France | 14.20 | 19.76 | Vietnam | 4.93 | 26.93 |
| Georgia | 2.97 | 23.37 | Slovenia | 3.52 | 20.88 |
| Germany | 16.90 | 22.47 | Spain | 26.91 | 17.27 |
| Greece | 3.35 | 26.90 | Sweden | 2.83 | 19.23 |
| Hong Kong (China) | 15.35 | 29.32 | Switzerland | 15.78 | 22.34 |
| Hungary | 7.61 | 22.57 | Thailand | 6.63 | 27.02 |
| Iceland | 0.94 | 17.19 | United Arab Emirates | 9.41 | 31.05 |
| Indonesia | 15.22 | 41.12 | Turkey | 7.22 | 24.11 |
| Ireland | 5.37 | 22.70 | Ukraine | 1.46 | 22.20 |
| Italy | 12.95 | 23.69 | United Kingdom | 2.40 | 27.07 |
| Kosovo | 4.17 | 31.82 | United States | 8.92 | 25.83 |
| Kazakhstan | 3.06 | 32.07 | Uruguay | 26.19 | 25.53 |
| Jordan | 10.24 | 37.90 | B-S-J-Z ^*^(China) | 8.29 | 17.72 |
| Korea | 4.42 | 9.40 | Moscow Region (RUS) | 1.50 | 37.71 |
| Latvia | 3.47 | 35.43 | Tatarstan (RUS) | 1.12 | 36.97 |

^*^ B-S-J-Z refers to the four PISA participating China provinces: Beijing, Shanghai, Jiangsu, and Zhejiang.
