# Supplementary material for: Grade repetition and bullying victimization in adolescents: A global cross-sectional study of the Program for International Student Assessment (PISA) data from 2018
Source: PLoS Med. 2021 Nov 11;18(11):e1003846. doi: 10.1371/journal.pmed.1003846 (PMC8584722; doi:10.1371/journal.pmed.1003846)
Supplement: S7 Table — (DOCX) [file pmed.1003846.s007.docx]

S7 Table. Country-specific prevalence of any type of bullying victimization among promoted students and grade repeaters, weighted %

| Country/economy | Promoted students | Grade repeaters | Country/economy | Promoted students | Grade repeaters |
| --- | --- | --- | --- | --- | --- |
| Albania | 25.18 | 32.78 | Lithuania | 22.33 | 34.99 |
| Baku (Azerbaijan) | 35.12 | 60.26 | Luxembourg | 16.96 | 28.75 |
| Argentina | 30.52 | 36.85 | Macao (China) | 25.55 | 30.48 |
| Australia | 28.82 | 41.22 | Malta | 30.86 | 50.61 |
| Austria | 21.95 | 31.26 | Mexico | 21.98 | 32.26 |
| Belgium | 16.22 | 24.19 | Moldova | 23.49 | 42.58 |
| Bosnia and Herzegovin | 24.81 | 50.12 | Montenegro | 24.68 | 49.59 |
| Brazil | 26.01 | 35.75 | Morocco | 38.16 | 51.26 |
| Brunei Darussalam | 48.33 | 63.50 | Netherlands | 11.39 | 15.92 |
| Bulgaria | 33.07 | 49.24 | New Zealand | 31.18 | 38.16 |
| Belarus | 18.35 | 30.83 | Panama | 29.87 | 43.54 |
| Canada | 24.84 | 32.95 | Peru | 21.46 | 28.86 |
| Chile | 22.50 | 28.85 | Philippines | 63.00 | 72.34 |
| Taiwan (China) | 13.16 | 28.98 | Poland | 26.16 | 30.57 |
| Colombia | 28.78 | 37.94 | Portugal | 10.34 | 23.52 |
| Costa Rica | 22.52 | 28.77 | Qatar | 30.99 | 43.54 |
| Croatia | 18.03 | 27.32 | Romania | 33.18 | 47.82 |
| Czech Republic | 28.91 | 47.60 | Russian Federation | 36.51 | 40.01 |
| Denmark | 21.08 | 31.25 | Saudi Arabia | 28.68 | 37.54 |
| Dominican Republic | 39.20 | 55.98 | Serbia | 25.25 | 43.46 |
| Estonia | 25.22 | 32.20 | Singapore | 25.67 | 32.13 |
| Finland | 17.54 | 21.68 | Slovak Republic | 27.14 | 48.78 |
| France | 17.94 | 30.79 | Vietnam | 25.94 | 46.03 |
| Georgia | 22.80 | 42.01 | Slovenia | 20.20 | 39.33 |
| Germany | 21.05 | 29.46 | Spain | 14.28 | 25.42 |
| Greece | 26.45 | 40.03 | Sweden | 18.91 | 30.10 |
| Hong Kong (China) | 29.27 | 29.60 | Switzerland | 20.55 | 31.92 |
| Hungary | 21.32 | 37.68 | Thailand | 25.78 | 44.59 |
| Iceland | 16.97 | 40.42 | United Arab Emirates | 29.58 | 45.23 |
| Indonesia | 39.75 | 48.72 | Turkey | 23.06 | 37.57 |
| Ireland | 22.37 | 28.39 | Ukraine | 21.91 | 41.72 |
| Italy | 22.45 | 32.01 | United Kingdom | 26.72 | 41.21 |
| Kosovo | 31.16 | 47.05 | United States | 25.20 | 32.25 |
| Kazakhstan | 31.45 | 51.64 | Uruguay | 22.10 | 35.19 |
| Jordan | 36.12 | 53.53 | B-S-J-Z ^*^(China) | 17.15 | 24.12 |
| Korea | 9.21 | 13.40 | Moscow Region (RUS) | 37.42 | 56.55 |
| Latvia | 34.73 | 55.14 | Tatarstan (RUS) | 36.85 | 48.20 |

^*^ B-S-J-Z refers to the four PISA participating China provinces: Beijing, Shanghai, Jiangsu, and Zhejiang.
