# Supplementary material for: Grade repetition and bullying victimization in adolescents: A global cross-sectional study of the Program for International Student Assessment (PISA) data from 2018
Source: PLoS Med. 2021 Nov 11;18(11):e1003846. doi: 10.1371/journal.pmed.1003846 (PMC8584722; doi:10.1371/journal.pmed.1003846)
Supplement: S8 Table — (DOCX) [file pmed.1003846.s008.docx]

S8 Table. Country-specific association between grade repetition and any type of bullying victimization

| Country/economy | Odds ratio (95%CI, p value) | |
| --- | --- | --- |
|  | Crude | Adjusted ^#^ |
| Albania | 1.45(0.95-2.20, 0.083) | 1.01(0.67-1.53, 0.956) |
| Baku (Azerbaijan) | 2.80(1.91-4.11, <0.001) | 2.60(1.68-4.03, <0.001) |
| Argentina | 1.33(1.14-1.55, <0.001) | 1.12(0.95-1.32, 0.174) |
| Australia | 1.73(1.44-2.08, <0.001) | 1.59(1.29-1.96, <0.001) |
| Austria | 1.62(1.22-2.14, 0.001) | 1.42(1.09-1.85, 0.011) |
| Belgium | 1.65(1.43-1.90, <0.001) | 1.59(1.36-1.87, <0.001) |
| Bosnia and Herzegovin | 3.05(1.97-4.72, <0.001) | 2.58(1.54-4.33, <0.001) |
| Brazil | 1.58(1.40-1.80, <0.001) | 1.35(1.16-1.57, <0.001) |
| Brunei Darussalam | 1.86(1.55-2.24, <0.001) | 1.53(1.25-1.88, <0.001) |
| Bulgaria | 1.96(1.43-2.70, <0.001) | 1.47(0.90-2.39, 0.126) |
| Belarus | 1.98(1.15-3.42, 0.015) | 1.46(0.80-2.64, 0.212) |
| Canada | 1.49(1.25-1.77, <0.001) | 1.41(1.17-1.69, 0.001) |
| Chile | 1.40(1.16-1.69, 0.001) | 1.44(1.19-1.74, <0.001) |
| Taiwan (China) | 2.69(1.40-5.18, 0.003) | 2.17(1.03-4.55, 0.042) |
| Colombia | 1.51(1.34-1.71, <0.001) | 1.35(1.20-1.52, <0.001) |
| Costa Rica | 1.39(1.20-1.61, <0.001) | 1.33(1.14-1.54, <0.001) |
| Croatia | 1.71(1.00-2.91, 0.049) | 1.55(0.86-2.82, 0.145) |
| Czech Republic | 2.23(1.63-3.06, <0.001) | 1.92(1.40-2.63, <0.001) |
| Denmark | 1.70(1.19-2.43, 0.004) | 1.49(0.99-2.26, 0.058) |
| Dominican Republic | 1.97(1.54-2.53, <0.001) | 1.89(1.41-2.53, <0.001) |
| Estonia | 1.41(0.95-2.09, 0.089) | 1.24(0.83-1.84, 0.296) |
| Finland | 1.30(0.89-1.89, 0.166) | 1.13(0.74-1.73, 0.575) |
| France | 2.04(1.64-2.52, <0.001) | 1.78(1.40-2.27, <0.001) |
| Georgia | 2.45(1.75-3.44, <0.001) | 1.66(1.03-2.67, 0.038) |
| Germany | 1.57(1.18-2.09, 0.003) | 1.41(1.06-1.89, 0.020) |
| Greece | 1.86(1.22-2.82, 0.004) | 1.12(0.67-1.87, 0.664) |
| Hong Kong (China) | 1.02(0.86-1.20, 0.850) | 0.96(0.80-1.16, 0.681) |
| Hungary | 2.23(1.73-2.88, <0.001) | 1.79(1.30-2.45, <0.001) |
| Iceland | 3.32(1.41-7.80, 0.006) | 1.96(0.72-5.34, 0.188) |
| Indonesia | 1.44(1.20-1.72, <0.001) | 1.40(1.15-1.72, 0.001) |
| Ireland | 1.38(1.02-1.85, 0.035) | 1.29(0.94-1.76, 0.113) |
| Italy | 1.63(1.27-2.08, <0.001) | 1.26(0.95-1.67, 0.102) |
| Kosovo | 1.96(1.42-2.71, <0.001) | 1.90(1.28-2.84, 0.002) |
| Kazakhstan | 2.33(1.79-3.02, <0.001) | 2.05(1.50-2.80, <0.001) |
| Jordan | 2.04(1.64-2.53, <0.001) | 1.56(1.26-1.94, <0.001) |
| Korea | 1.53(0.99-2.34, 0.054) | 1.39(0.90-2.13, 0.133) |
| Latvia | 2.31(1.63-3.28, <0.001) | 1.86(1.29-2.67, 0.001) |
| Lithuania | 1.87(1.18-2.98, 0.009) | 1.23(0.75-2.00, 0.406) |
| Luxembourg | 1.98(1.69-2.31, <0.001) | 1.81(1.54-2.12, <0.001) |
| Macao (China) | 1.28(1.10-1.48, 0.002) | 1.20(1.02-1.40, 0.025) |
| Malta | 2.29(1.61-3.28, <0.001) | 1.84(1.26-2.69, 0.002) |
| Mexico | 1.69(1.19-2.41, 0.004) | 1.59(1.13-2.23, 0.009) |
| Moldova | 2.41(1.59-3.66, <0.001) | 1.93(1.20-3.10, 0.007) |
| Montenegro | 3.00(1.77-5.11, <0.001) | 2.32(1.27-4.24, 0.007) |
| Morocco | 1.70(1.41-2.06, <0.001) | 1.56(1.23-1.97, <0.001) |
| Netherlands | 1.47(1.11-1.96, 0.009) | 1.39(1.02-1.90, 0.036) |
| New Zealand | 1.36(1.05-1.77, 0.021) | 1.31(0.99-1.72, 0.055) |
| Panama | 1.81(1.39-2.36, <0.001) | 1.77(1.29-2.43, 0.001) |
| Peru | 1.49(1.14-1.94, 0.004) | 1.39(1.03-1.86, 0.029) |
| Philippines | 1.54(1.33-1.78, <0.001) | 1.36(1.17-1.58, <0.001) |
| Poland | 1.24(0.71-2.19, 0.445) | 1.10(0.61-1.99, 0.752) |
| Portugal | 2.67(2.24-3.18, <0.001) | 2.30(1.88-2.82, <0.001) |
| Qatar | 1.72(1.55-1.91, <0.001) | 1.54(1.37-1.73, <0.001) |
| Romania | 1.85(1.25-2.74, 0.003) | 1.14(0.72-1.82, 0.575) |
| Russian Federation | 1.16(0.78-1.73, 0.461) | 0.88(0.56-1.38, 0.579) |
| Saudi Arabia | 1.49(1.20-1.86, <0.001) | 1.25(0.98-1.59, 0.072) |
| Serbia | 2.28(0.85-6.11, 0.102) | 1.63(0.49-5.45, 0.422) |
| Singapore | 1.37(0.92-2.04, 0.118) | 1.22(0.79-1.88, 0.367) |
| Slovak Republic | 2.56(2.04-3.21, <0.001) | 1.89(1.45-2.46, <0.001) |
| Vietnam | 2.43(1.83-3.24, <0.001) | 2.14(1.57-2.92, <0.001) |
| Slovenia | 2.56(1.37-4.79, 0.004) | 1.92(0.82-4.51, <0.132) |
| Spain | 2.05(1.86-2.25, <0.001) | 1.73(1.57-1.92, <0.001) |
| Sweden | 1.85(1.26-2.71, 0.002) | 1.60(1.05-2.45, 0.030) |
| Switzerland | 1.81(1.44-2.28, <0.001) | 1.69(1.31-2.20, <0.001) |
| Thailand | 2.32(1.85-2.91, <0.001) | 1.71(1.34-2.18, <0.001) |
| United Arab Emirates | 1.97(1.72-2.25, <0.001) | 1.54(1.31-1.82, <0.001) |
| Turkey | 2.01(1.53-2.63, <0.001) | 1.49(1.14-1.95, 0.004) |
| Ukraine | 2.55(1.59-4.10, <0.001) | 1.53(0.84-2.79, 0.160) |
| United Kingdom | 1.92(1.33-2.78, 0.001) | 2.16(1.40-3.33, 0.001) |
| United States | 1.41(1.08-1.85, 0.013) | 1.32(0.99-1.76, 0.058) |
| Uruguay | 1.91(1.58-2.32, <0.001) | 1.76(1.42-2.18, <0.001) |
| B-S-J-Z ^*^(China) | 1.54(1.12-2.11, <0.001) | 1.37(0.99-1.89, 0.058) |
| Moscow Region (RUS) | 2.18(0.88-5.39, 0.092) | 2.41(0.79-7.41, 0.122) |
| Tatarstan (RUS) | 1.60(0.83-3.05, 0.156) | 1.04(0.51-2.11, 0.910) |

^#^Models adjusted sex, age group, migrant status, school type, economic, social and culture status, and parental emotional support. ^*^ B-S-J-Z refers to the four PISA participating China provinces: Beijing, Shanghai, Jiangsu, and Zhejiang.
