# Supplementary material for: Grade repetition and bullying victimization in adolescents: A global cross-sectional study of the Program for International Student Assessment (PISA) data from 2018
Source: PLoS Med. 2021 Nov 11;18(11):e1003846. doi: 10.1371/journal.pmed.1003846 (PMC8584722; doi:10.1371/journal.pmed.1003846)
Supplement: Attachment — Submitted filename: Rebuttal letter.docx [file pmed.1003846.s009.docx]

Dear Dr. Caitlin Moyer,

We are pleased to submit the revised version of the manuscript titled “Grade repetition and bullying victimization in teenagers: a global cross-sectional study” to PLOS Medicine.

We worked on addressing all requests and comments from the editors and reviewers and incorporating them into our manuscript where suitable, including more analysis and additional tables.

We thank the editors and reviewers for all their requests and comments, which allowed us to clarify several points and improve our manuscript. And you will find below a point-by-point response to the concerns raised by the editors and reviewers.

Besides, we updated the authors’ information because the first affiliation (Shanghai Institute of Planned Parenthood Research) has been renamed Shanghai Institute for Biomedical and Pharmaceutical Technologies.

Please don't hesitate to contact us if you have further questions.

Sincerely yours,

Dr. Qiru Su and Dr. Xiayun Zuo

**Requests from the editors:**

1. Abstract: Background: Line 24 (and throughout): Please refer to low or middle income countries rather than "developing countries" and high income countries rather than "developed" countries.

Response: We revised the background part in Abstract and checked throughout of the manuscript as you suggested.

2. Abstract: Methods and findings: Please clarify the age of the students (at line 32 this is indicated as 15 years, but at line 41 a range of 15.08-16.33 years is given). number of participants, years during which the study took place, length of follow up, and main outcome measures.

Response: In PISA related publications,“15-year-olds” and "15-year-old students" are used as shorthand for the PISA target population.

In practice, the target population is students aged between 15 years and 3 (complete) months and 16 years and 2 (complete) months at the beginning of the assessment period, and who were enrolled in an educational institution that they were attending full-time or part-time.

Since the majority of the PISA target population is made up of 15-year-olds, the target population is often referred to as 15-year olds.[1] We added this in Study design and participants part to minimized the potential misunderstanding.

We also clarified the number of participants, years during which the study took place, length of follow up, and main outcome measures as you suggested.

Reference:

[1] PISA 2015: A first look at Australia’s results. https://research.acer.edu.au/cgi/viewcontent.cgi?article=1021&context=ozpisa

3. Abstract: Conclusions: Line 57-58: Here, and throughout the manuscript, please avoid statements implying causality of the results. “These findings provide robust evidence for the negative effect of grade repetition on bullying victimization.”

Response: We removed the statements implying causality of the results here and throughout the manuscript as you suggested.

4. Author summary: Please format the author summary with bullet points rather than paragraph form (2-3 points per section). Please revise the final statement to avoid overstating what can be concluded from your study: “Globally the policy of grade repetition is harmful to students’ health and wellbeing in some way regarding to school bullying.”

Response: We formatted the author summary with bullet points and revised the final statement as you suggested.

5. Methods: Line 139-142: Please at least briefly mention how schools and students were selected for inclusion: “A two stage sampling procedure was adopted within each country/economy. In the first stage, a representative sample of at least 150 schools was selected, then roughly 42 students were selected from each school [22]. At line 149-150, please clarify this statement further: “The threshold for an acceptable participation rate after replacement was 85% [22].”
Response: We briefly described how schools and students were selected for inclusion as you suggested in Study design and participants. We rephased the statement on PISA participation rates (including schools and students), incorporated them into how schools and students were selected.

6. Methods: LIne 165-166: make sure that the Methods section transparently describes when analyses were planned, and when/why any data-driven changes to analyses took place (with rationale).

Response: There was no documented analytical protocol to be followed on addressing our specific research question in PISA related documents, and no any data-driven changes to analyses took place. We transparently described the analysis plan, data analysis process and no data-driven changes in Methods / Statistical analysis section as you suggested.

7. Methods: Line 198: Please revise if this should be “Are you female or male?” in this question.

Response: It was a typo and has been revised, thanks for pointing that out.

8. Methods: Line 217-219: Please clarify the measures used to derive ESCS.

Response: We added more information on measures used to derive ESCS as you suggested.

9. Methods: Line 220-225: Please provide more detail on how the parental support measure was derived.
Response: The derived variable was constructed using item response theory scaling methodology. We added the information as you suggested.

10. Methods: If possible, please include the PISA questions used to obtain the data on measures and covariates as a supporting information file.

Response: We included measures related questions in PISA 2018 as a supporting information file (S3 Table) as you suggested.

11. Results: Line 258-259: Please clarify this sentence, as the footnote of Table 2 suggests that there are differences: “There were no statistically significant differences in the distribution of individual characteristics between boys and girls.”

Response: “no statistically significant differences” refers to “individual characteristics” as described in this sentence and in Table 1. We removed this sentence because of potential misunderstanding.

12. Results: Line 260-261: It may be useful to provide a range here for prevalence of grade repetition.

Response: Thanks for your suggestion. We provided the 95%CI for prevalence of grade repetition and bullying victimization as you suggested.

13. Results: Line 290-292: Please provide P values in addition to 95% CIs for sensitivity analyses.

Response: Thanks for your suggestion. We provided the P values in addition to 95% CIs for sensitivity analyses as you suggested.

14. Discussion: Line 302-304: Please avoid the use of causal language, here and throughout the Discussion.

Response: We revised and removed the causal language here and throughout the manuscript as you suggested here and above in the Abstract.

15. Discussion: Line 338: “The effect of grade repetition on bullying victimization is negative in our study.” Please avoid implying causality in this sentence.

Response: We revised and removed the causal language here throughout the manuscript as you suggested here and aforementioned comment #3 and #14.

16. Discussion: Line 381: Please remove the phrase “if causal” from this sentence, as your study cannot inform on the causal nature of this relationship.

Response: We removed the phrase “if causal” as you suggested.

17. Discussion: Line 383-384: Please revise to avoid causal implications: “Our study provides robust evidence for the negative consequence of grade repetition on bullying victimization.”

Response: We revised and removed the causal language here throughout the manuscript as you suggested here and aforementioned comment #3, #14, #15 and #16.

18. Discussion: Line 393-396: Please revise, interpreting the study based on the results presented in the abstract, emphasizing what is new without overstating your conclusions. “Our findings confirm that grade repetition is associated with bullying victimization and highlight that social promotion is a better policy practice than grade retention regarding to bullying victimization although both practices are inefficient 396 to improve educational outcomes of students.”
Response: We revised the conclusion, and emphasized our findings without overstating our conclusions.
19. Please provide titles and legends for each individual table and figure in the Supporting Information.
Response: We provided titles for each individual table in the Supporting Information. We did not have figure in the Supporting file.

20. Table 2 and Table 3: Please provide the statistical results (95% CIs and P values ) that are missing from the Table.

Response: Thanks for your suggestion. Because All P values in Table 2 and 3 are less than 0.001, we provided the P values in the note below the two Tables. The statistical results in Table 2 and 3 are Pearson Chi-Square values and corresponding P values (as listed in below Table for example). We don’t think it provides more information for readers, hence we didn’t change the frame of the two Tables. Please let us know if you have any comments.

Table 2 Prevalence of grade repetition and bullying victimization among girls and boys, n (%)

|  | Girls (n=234,218) | Boys (n=230,928) | Total (n=465,146) | χ² | P value |
| --- | --- | --- | --- | --- | --- |
| Grade repetition |  |  |  |  |  |
| Retained students | 19,019(9.53) | 27,754(15.01) | 46,773(12.26) | 322.62 | <0.001 |
| Types of Bullying |  |  |  |  |  |
| Other students left me out of things on purpose. | 23,733(12.61) | 28,677(14.85) | 52,410(13.72) | 58.67 | <0.001 |
| Other students made fun of me. | 28,905(15.67) | 41,594(20.22) | 70,499(17.92) | 140.28 | <0.001 |
| I was threatened by other students. | 13,304(7.37) | 26,432(12.77) | 39,736(10.05) | 271.80 | <0.001 |
| Other students took away or destroyed things that belonged to me. | 15,465(9.37) | 28,862(14.74) | 44,327(12.03) | 286.71 | <0.001 |
| I got hit or pushed around by other students. | 13,175(7.35) | 28,724(13.60) | 41,899(10.45) | 393.48 | <0.001 |
| Other students spread nasty rumours about me. | 25,201(12.40) | 32,803(15.66) | 58,004(14.01) | 87.33 | <0.001 |
| Any form of above | 53,513(27.06) | 70,59(33.61) | 124,103(30.32) | 200.48 | <0.001 |

21. S3 Table: Please provide individual P values for each result.

Response: We provided individual P values, but when responding to reviewer 2, we deleted the table. In other supplement tables, we also provided individual P values for each result as you suggested.

**Comments from the reviewers:**

Reviewer #1: This study aims to investigate the global association of grade repetition with bullying victimization in a large-scale school-based cross-sectional study.

Comments:

"All participants in Japan, Malaysia and Norway were excluded because no students in the three countries have ever repeated a grade (n=18,033). "
Can the authors please assess the bullying in these three countries for comparison of prevalence/frequency, given no grade repetition?

Response: We estimated the the bullying in these three countries as you suggested. The weighted prevalence of any type of bullying victimization in Japan, Malaysia and Norway was 17.27%, 35.74% and 18.82% respectively. As we know, the prevalence of bullying varies considerably across the globe, hence we compared the prevalence of bullying victimization between retained and non-retained students within the counties that have policy of grade repetition after controlling the clustering effects of countries using Stata svy command.

"Last, the participants without the data on grade repetition or school bullying (n=111,017) were excluded from the analysis"
Can the authors please discuss whether these excluded participants can be considered to be missing at random (MAR)?

Response: Thanks for the suggestion. It’s essential to understand the missing data mechanisms before choosing an approach. Missing at random(MAR) means the likelihood of a value to be missing depends on other observed variables, then multiple imputation can be utilized to ‘recover’ the missing data based on observed data.

We used complete case analysis in our study based on following two considerations:

1) The participants accidentally skipped questions in our opinion is the key reason behind the missing data. We hence tend to believe the information on grade repetition and school bullying is missing completely at random (MCAR), and the completed observations can be treated as a random subsample of the population. Complete case analysis under MCAR this does not lead to bias of the results, nor decreases the power of the test considerably given the huge sample size in our study.

2) The grade repetition and school bullying are outcome data. Complete case analysis is more appropriate because imputed outcome data can lead to misleading results [2-4].

Reference:

[2]Papageorgiou G, Grant SW, Takkenberg JJM, Mokhles MM. Statistical primer: how to deal with missing data in scientific research? Interact CardioVasc Thorac Surg 2018;27:153–8.

[3]Von Hippel PT. Regression with missing Ys: an improved strategy for analyzing multiply imputed data. Sociol Methodol 2007; 37:83–117.

[4]Little RJA. Regression with missing X’s: a review. J Am Stat Assoc 1992;87:1227–37.

The authors have followed STROBE and suitably included the associated checklist with the supplementary material.

Response: Thanks for your comments.

"Participants were categorized as retained students if they answered "Yes" to any of these three questions, and promoted students if they didn't have the experience with repeating a grade at school before the survey."
Did the authors consider keeping "Yes, once" or "Yes, twice or more" as separate groups for the analysis, to explore the possibility of a dose-response relationship?

Response: Thanks for the suggestion. The global weighted percentage of “Yes, twice or more” for repeating a grade at ISCED 1, ISCED 2 and ISCED 3 was 1.02%, 1.05% and 0.17%, respectively. Given the low percentage of grade repetition, we didn’t explore the possibility of a dose-response relationship in our study.

"The response options were "never or almost never", "a few times a year", "a few times a month", or "once a week or more". For each of the six experiences mentioned above, we identified bullying victimization by the responses of "a few times a month" or "once a week or more" [25, 26]. Besides, we combined the six experiences into a single indicator, "any type of bullying victimization", if the participants reported being bullied frequently (at least a few times a month) in any type".
Did the authors complete any sensitivity analyses looking at different ways to dichotomise bullying victimisation? Furthermore, did they consider keeping all four categorical outcomes within the analysis?

Response: Thanks for the suggestion. We didn’t look at different ways to dichotomies bullying victimization. With regard to the cutoff point (response alternative) used in deciding when a respondent is considered being bullied, we used “a few times a month or more” , as “2 or 3 times a month or more” has been used widely in large numbers of large-scale research studies on bullying as a lower boundary.

Prof. Dan A. Olweus, pioneer researcher of bullying, wrote: “After a detailed discussion of conceptual and strategic arguments and several empirical analyses, it was decided that individuals who respond “2 or 3 times a month” or more often can be classified (for research purposes) as “being bullied” [5] and “Prevalence estimates derived in this way can be conveniently obtained, have a reasonably well-defined meaning, can be easily understood by users, and can be reproduced unambiguously by different researchers/administrators and at different times [6].”

Many national representative studies aimed to assess relative effects of bullying victimization on health also selected this cutoff point to represent regular bullying to distinguish between young people who were meaningfully involved in bullying and those who were not [7].

Also, we generated a composite score as reviewer #2 suggested, to explore the association of grade repetition with victimization degree (score).

Reference:

[5] Olweus, D. (2017). Cyberbullying: A critical overview. In B. J. Bushman (Ed.), Aggression and Violence: A Social Psychological Perspective (pp. 225- 240) New York: Routledge.

[6] Solberg, M.E. and Olweus, D. (2003), Prevalence estimation of school bullying with the Olweus Bully/Victim Questionnaire. Aggr. Behav., 29: 239-268.
[7] Przybylski, A. K., & Bowes, L. (2017). Cyberbullying and adolescent well-being in England: a population-based cross-sectional study. The Lancet Child & Adolescent Health, 1(1), 19–26.

"Demographic characteristics assessed included sex, age group, immigration status, school type, economic, social and culture status (ESCS), and perceived parents' emotional support".
Did the authors consider including BMI, disability, ethnicity, or urban/rural school location as other potential confounders in their analysis?
Response: Thanks for the suggestion. PISA 2018 did include the measure on BMI, but only in a few countries, hence more than 95% (exactly 95.16%) of the participants in the final sample didn’t report the information (height and weight). PISA 2018 dataset didn’t include the information on disability, ethnicity, or urban/rural school location as you mentioned. We acknowledged these unmeasured potential confounders in the third limitation in our study.

"As suggested by a study of PISA 2009 [2], we generated a dichotomous variable "age group" with 1 indicating the age is below the country average age, and 2 indicating the age is at or above the country average age".
Did the authors consider completing additional analyses treating age as a continuous variable?

Response: Thanks for the suggestion. The target population in PISA is students aged between 15 years and 3 (complete) months and 16 years and 2 (complete) months. The age ranged 15.08 to 16.33 years in our final sample.

Given the age range is very narrow (the majority of the PISA target population is made up of 15-year-olds), we didn’t treat the age as a continuous variable.

"Finally, we adopted multivariable logistic regression models to estimate the strength, direction, and statistical significance of the relations linking experience with grade repetition to each type of bullying victimization by sex, adjusting the participating country ID and the measured control variables aforementioned in covariates assessment".
Did the authors consider applying hierarchical or multilevel modelling to better allow for clustering by country differences? Furthermore, there may be school differences within countries?

Response: Thanks for the reminder. The international publicly available data for PISA are delivered with replicate weights, which implicitly reflect design features. Also, PISA data files contain no information about school-level weight, only student-level weight (final trimmed nonresponse-adjusted student weight).

As described in the Statistical analysis part, in this survey data, we considered the complex survey sampling design and sampling weight in all analyses using Stata *svy*: prefix commands. Given that we are interested in the global association of grade repetition with bullying victimization, not estimating the county-level impacts, we adjusted the country differences and school differences with PSU (egen PSU= group (countryID SchoolID)) in Stata *svyset* command (declare survey design for dataset) as separate samples were taken in each school and each country.

"The recently proposed E-value quantitates the minimum strength of association with both the exposure and the outcome which would be required of an unmeasured confounder to fully explain away the observed effect [30, 32]."
This is a novel and useful approach for estimating the possible strength of impact of unknown confounding on the study inferences. Can the authors please clarify within the manuscript if this analysis considers one confounder at a time, or multiple confounders working together?

Response: Thanks for the suggestion. E-value is a post-estimation after multivariate logistic/linear regression models, hence it considers multiple confounders working together, we revised the manuscript to clarify this as you suggested.

The authors are correct to state that the study design means that causality cannot be inferred.
Therefore, the evidence provided from this study cannot determine whether grade repetition increases the chance of bullying, or bullying victimisation increases chance of grade repetition.
To introduce analyses that take into account the timing of events is a sensible idea, that might help uncover the direction of association that is observed in the study findings: "The reference period of being bullied (past 12 months before PIAS 2018) and repeating a grade at ISCED3 may overlap. To establish the temporal relationship between grade repetition and bullying victimization, we further repeated the analyses of the associations of repeating a grade at ISCED1 or ISCED2 with the risk of being bullied."
However, this does not allow for occurrences of bullying prior to the last 12 months, which is not captured by the data.

The authors state in the results that: "As a sensitivity analysis we also evaluated associations of repeating a grade at ISCED1 or ISCED2 with the risk of being bullied to make sure exposure (grade repetition) preceded outcomes (bullying victimization), and found similar results (S3 Table). For instance, the multivariable adjusted OR (95% 291 CI) for bullying victimization in any type comparing promoted students to retained students in all students was 1.44(1.34-1.55); in girls: 1.51(1.34-1.70); and in boys: 1.40(1.29-1.52). "
This is misleading as in fact this analysis does not 'make sure exposure (grade repetition) preceded outcomes (bullying victimization)'. Occurrences of bullying victimisation preceding ISCED1 or ISCED2 have not been captured in the data included within this analysis. Even more entwined, the relationship between grade repetition and bullying victimisation could be iterative in nature.

Response: Thanks for the comments. Yes, grade repetition is of reciprocal causation with bullying victimization. Grade repetition may increase the chance of being bullied, and bullying victimization also increases the chance of repeating a grade.

The data on bullying victimization receding ISCED1 or ISCED2 are unavailable and temporal direction is not ensured by our analysis. We removed this sensitivity analysis because it was misleading as you suggested.

Furthermore, the conclusions that ""Globally the policy of grade repetition is harmful to students' health and wellbeing in some way regarding to school bullying" cannot and should not be inferred from the study outcomes found from this research.

Response: Thanks for the comments. The same concern is also raised by the editors. We removed and revised to avoid overstating our results and any causal language here and throughout the manuscript.

The authors acknowledge that: "The major limitation is this study only included students attended the school, which may be subject to possible selection bias. In addition, the cross-sectional design hinders us from establishing causality between grade repetition and bullying victimization", but later state that "however, we also used a finer category of repeating a grade before ISCED3 to ensure grade reputation preceded bullying victimization, and further examined the temporal direction of the relationship between grade repetition and bullying victimization", which is inaccurate as temporal direction is not ensured by the analysis due to the outcome question asking about the last 12 months only.
Response: Thanks for the comments. We removed the statement in limitation part because it is inaccurate and misleading as you mentioned here and previously.

Similarly, the authors go on to state that: "Our findings, if causal and generalizable to broader age groups, may have significant implications for the wellbeing of 1.3 billion school-age children and adolescents around the world in an era embracing evidence-based interventions [47]. Our study provides robust evidence for the negative consequence of grade repetition on bullying victimization. The observed link with grade repetition and bullying victimization implies that the widespread educational policy of grade repetition may partly contribute to the differences in bullying victimization, and adds to the evidence against the policy of grade repetition."
This needs to be completely reworded, with the authors applying far more caution and care with their stated study implications and conclusions. Causality cannot be inferred by this analysis, and therefore causality should not be implied in the interpretation of the outcomes.
Response: Both you and the editors suggested to avoid statements implying causality of the results.

We removed and revised to avoid overstating our results and any causal language here and throughout the manuscript.

And again, "Our findings... highlight that social promotion is a better policy practice than grade retention regarding to bullying victimization although both practices are inefficient to improve educational outcomes of students."
This statement is not reflective of the evidence presented here.
Response: Thanks for the comments, the editors also mentioned this in #18. We revised the conclusion, and emphasized our findings without overstating our conclusions.

Reviewer #2: I like the idea of looking at grade retention and bullying victimization using the PISA data. I have a few concerns to consider.
1. I would not look at bullying victimization by type. We have shown as have others that what matters for outcomes is severity and not form (see Haltigan & Vaillancourt, 2018). You also improve measurement precision by creating a composite score as suggested by Vaillancourt et al., 2010.

Response: Yes, different types of bullying victimization tend to co-occur, indicating that specific types have a lot in common and are unlikely to have distinct factors. We agree that victimization degree (severity) rather than type (form) matters for outcomes [8], for example it can be used to identify the most vulnerable children for intervention. In the present study, the bullying victimization is the outcome, and retained students are supposed to have decreased risk of specific victimization type (physical bullying) in existing studies.

We added a derived composite score as you suggested [9] to explore the association of grade repetition with levels of bullying victimization.

Reference:

[8] Haltigan, J.D., Vaillancourt, T. (2018). The Influence of Static and Dynamic Intrapersonal Factors on Longitudinal Patterns of Peer Victimization through Mid-adolescence: a Latent Transition Analysis. J Abnorm Child Psychol 46, 11–26.

[9] Wang, W., Brittain, H., McDougall, P., & Vaillancourt, T. (2016). Bullying and school transition: Context or development? Child Abuse & Neglect, 51, 237–248.

2. I would concentrate my efforts on looking at grade retention by bullying victimization by country. You have this in your S2 table; it should replace your table on type.

Response: Thanks for your suggestion. As the outcome is bullying victimization, and the exposure is grade retention in the present manuscript, we added country-specific information (prevalence and score) of bullying victimization by grade retention as you suggested (S5 table-S6 table, S8 table-S9 table).

3. The implied direction is that being retained impacts bullying but it is possible that being bullied impact grades which impacts retention. There are longitudinal studies supporting both pathways bullied to poor grades and poor grades to being bullied. This needs to be addressed in the manuscript.

Response: Yes, grade repetition is of reciprocal causation with bullying victimization. We acknowledged it in 2nd limitation as you and reviewer #2 suggested.

4. Boys are more likely than girls to be retained than girls and to be bullied. I would look at this as a 3-way interaction. Maybe even a 4-way as per suggestion #2.

Response: We analyzed interaction effects of grade retention and sex on bullying victimization measured by type and score respectively (S4 table).

5. There are a number of typos that need to be addressed (example lines 107-108). Avoid using contractions like isn't or didn't.
Response: Thanks for your comments. We revised the contractions (eg. isn’t, didn’t) here and throughout the manuscript.

Reviewer #3: The manuscript, Grade repetition and bullying victimization in teenagers: A global cross-sectional study, examines the association between a history of grade retention and experiences of bullying victimization in the past year for adolescents with an average age of about 15.8 years in 74 countries around the world. The study addresses a relatively neglected area of grade retention research by examining its association with bullying victimization. Strengths and weaknesses include the large, global sample. Strengths include the inclusion of so many countries around the world with a large sample size. Weaknesses include the averaging of the experiences of adolescents from such diverse countries with different cultures related to school experiences, peer relationships, and gender. The paper also would benefit from significant copy editing due to numerous typos, run-on sentences, and errors in word selection and grammar.
Response: Thanks for your comments. We performed more analysis by country as you suggested to explore the association among diverse countries with different cultures (S7 table and S10 table). We also revised the manuscript thoroughly to minimize the language issue.

The authors build their study rationale based on a lack of empirical studies relating grade retention and bullying victimization. They touch on some reasons why other researchers have explained associations or no associations between grade retention and bullying victimization. However, the study is not guided by a theoretical orientation or explanation of the mechanisms why grade retention would be related to bullying victimization. I recommend authors add an orientation and then also attend to how this orientation would relate to various cultural contexts around the globe. Would associations differ in different cultural contexts? Is an analysis by country warranted? Why or why not?
Response: We added theoretical orientation in the background. We also conducted more analysis by country as you suggested (S5 Table – S10 Table). We put them in Supplementary file because the main aim of the present study was to map an overall picture of the association of grade repetition with bullying victimization across the PISA participating countries.

The associations differed in a few cultural contexts, among the 74 countries and economies, the association was statistically non-significant in 28 countries using prevalence of victimization, and in 16 when using victimization score. However, when examining the prevalence and score of victimization, we found that retained students reported both higher prevalence and score than their peers did within each country.

At the bottom of page 10, the authors wrote, "Finally, we adopted multivariable logistic regression models to estimate the strength, direction, and statistical significance of the relations linking experience with grade repetition to each type of bullying victimization by sex, adjusting the participating country ID and the measured control variables aforementioned in covariates assessment." I was unclear how the authors used the participating country ID in the analysis. I am also curious why authors did not report the full results of the logistic regression in terms of the association of control variables to bullying victimization.

Response: Participating country ID refers to the numeric variable named “cntryid” and labelled “Country Identifier” in PISA 2018 dataset. The cntryid was treated as a dummy variable in the models. We prefixed cntryid with i. (i. cntryid) to specify indicators for each level (category) of the variable, so Stata can treat this variable as a dummy variable [10-11]. We revised it as “…adjusting the country variable and the measured control variables aforementioned in covariates assessment.”

We only reported the main results. Given that the full results of the regression in terms the control variables are far too lengthy for the scope of this manuscript. There are 8*3=24 models in Table 4 to explore the global associations between grade repetition and bullying victimization. The models are much more in S7 table and S10 table in Supplementary file.

Reference:

[10] Factor variables. https://www.stata.com/features/overview/factor-variables/

[11] How do I create dummy variables? https://www.stata.com/support/faqs/data-management/creating-dummy-variables/

Authors state that their research goal is to understand the association between grade repetition and bullying victimization, but they also touch upon gender differences. It would be helpful for authors to move from a theoretical orientation to the status of empirical evidence for their purpose to a final, complete set of research questions and hypotheses. Authors examine overall bullying, single bullying experiences (measured and not latent constructs), and gender differences. They should explain also why a global average is best and why examining individual country contexts is not needed.
Response:

We rephrased our research questions and hypotheses as you suggested. We also examined the association within individual participating country contexts (S7 table and S10 table) as you suggested here and in the 3^rd^ comment, in addition to the global average.

In their discussion, authors wrote, "The effect of grade repetition on bullying victimization is negative in our study" but, in fact, grade repetition was positively related to bullying victimization (students who had been retained were more likely to experience bullying victimization). Also, authors should avoid using terms like "effect" that imply a causal association.

Response: The word “negative” means the experience of being repeating a grade is “bad” for students’ health. We revised the conclusion to clear up any misunderstandings. We also removed the causal language like "effect" as you and editors and reviewer #1 suggested.
