# Supplementary material for: Grade repetition and bullying victimization in adolescents: A global cross-sectional study of the Program for International Student Assessment (PISA) data from 2018
Source: PLoS Med. 2021 Nov 11;18(11):e1003846. doi: 10.1371/journal.pmed.1003846 (PMC8584722; doi:10.1371/journal.pmed.1003846)
Supplement: Attachment — Submitted filename: Rebuttal letter2.docx [file pmed.1003846.s010.docx]

Dear Dr. Caitlin Moyer,

We are pleased to submit the revised version of the manuscript titled “Grade repetition and bullying victimization in teenagers: A global cross-sectional study” (PMEDICINE-D-21-00390R3) for consideration in PLOS Medicine’s Special Issue: Global Child Health: From Birth to Adolescence and Beyond.

We thank the Senior and Reviewing Editors of PLOS Medicine, the Special Issue Guest Editors, and external Reviewers for the detailed and constructive comments. We worked on addressing the requests and comments raised by the editors and reviewers and incorporating them into our manuscript where suitable, including more analysis and a language review.

We thank the Editors and Reviewers for all their requests and comments, which allowed us to clarify several points and improve our manuscript. And you will find below a point-by-point response to the concerns raised by the editors and reviewers.

Please don't hesitate to contact us if you have further questions.

Sincerely yours,

Dr. Qiru Su and Dr. Xiayun Zuo

**Requests from the editors:**

1. Title: Please capitalize the first word of the subtitle: “Grade repetition and bullying victimization in teenagers: A global cross-sectional study”

Response: We capitalize the first word of the subtitle “A global cross-sectional study”.

2. Abstract: Methods and Findings: Please note the PISA assessment is administered to all students attending secondary education, with ages ranging from 15-16 years. Please clarify what is meant by “deprived victimization score” by briefly describing this is a composite score.

Response: We added the PISA 2018 assessment was in the students attending secondary education, with ages ranging from 15-16 years, and also clarified the “deprived” victimization score (a composite victimization score (the average of all six items)). The “deprived” is a typo, it should be derived.

3. Abstract: Methods and Findings: Line 38: Please note the potential confounders adjusted for in the analysis.

Response: We added the potential confounders adjusted in the Abstract as you suggested.

4. Abstract: Methods and findings: Line 48: Please briefly elaborate on the sex differences observed.

Response: Retained girls were at higher risks of being made fun of, being threatened, possessions were taken away, and being pushed around than retained boys. We elaborated the sex differences in Abstract as you suggested.

5. Abstract: Conclusions: Please clarify the final sentence, we suggest: “Sex differences in risk of experiencing bullying suggest that girls who repeat a grade may benefit from tailored interventions.” or similar.

Response: We revised the last sentence as you suggested.

6. Author summary: Please revise the authors summary. Please provide 2-3 single sentence bullet points under each of the following headings. Bullet points should be objective, brief, succinct, specific, accurate, and avoid technical language.

-Why Was This Study Done? Authors should reflect on what was known about the topic before the research was published and why the research was needed.

-What Did the Researchers Do and Find? Authors should briefly describe the study design that was used and the study’s major findings. Do include the headline numbers from the study, such as the sample size and key findings.

-What Do These Findings Mean? Authors should reflect on the new knowledge generated by the research and the implications for practice, research, policy, or public health. Authors should also consider how the interpretation of the study’s findings may be affected by the study limitations.

Response: We revised the bullet points under the three headings.

7. In text references: Please include a space between the word and the reference bracket. For multiple references listed within brackets, please do not include a space (for example [13,15]).

Response: We checked the references and revised accordingly.

8. Introduction: Line 76: Please change “Belgian” to “Belgium” and please revise to read “...while grade repetition has been discouraged strongly…”

Response: Thanks for pointing it out. We revised accordingly.

9. Methods: Line 154-155: From looking at the Figure 1 flowchart, it is not clear whether in these countries grade repetition does not occur, or whether the data were not collected.

Response: Grade repetition is non-existent in Japan, Korea and Norway. We rephased this sentence as “All participants in Japan, Malaysia, and Norway were excluded because these three countries do not allow for grade repetition and no students reported being retained (n=18,033)”.

10. Methods: Line 188: Please provide more information on the construction of the composite victimization score.

Response: We provided more information on how to construction of the composite victimization score as you suggested.

11. Methods: Line 216: Please define the abbreviation ESCS at the first point of use in the text.

Response: We did define the abbreviation ESCS at the first point of use in the beginning of the Covariates section.

12. Methods: Line 240: Please indicate any changes made to the analysis in response to peer reviewer comments (such as the implementation of the victimization composite score).

Response: We indicated the changes made to the analysis in response to peer reviewer comments in the first paragraph of Statistical analysis section.

13. Methods: Is it possible to include a supporting information file describing the average ages by country?

Response: We include a supporting information file (S4 Table) describing the average ages by country in Results section.

14. Results: Line 289: Please provide the exact p values for the sex-specific analyses describing victimization score differences for students who were retained compared to those who were promoted.

Response: We provided the exact p values for the sex-specific analyses as you suggested.

15. Results: Line 314: Please change “regress” to “regression” in this sentence. Please quantify the results from this analysis with 95% CIs and p values.

Response: Thanks for pointing it out. We changed “regress” to “regression” as you suggested. It’s inappropriate in our opinion to quantify the results with 95% CIs and p values because it a result summary of the 74 countries.

16. Discussion: Line 364-365: We suggest revising to “...may be a contributing factor to the relationship between grade repetition and bullying…” or similar to avoid causal implications.

Response: Thanks for pointing it out. We rephased the sentence as you suggested to avoid causal implications.

17. Discussion: Line 378: We suggest revising to “These findings suggest that girls may benefit from targeted attention in anti-bullying programs.” or similar.

Response: We revised the sentence as you suggested.

18. Discussion: Line 384-385: Please clarify what is meant by “...because the actual cross-national comparable data on bullying can only be collected by pictures.”

Response: Thanks for pointing it out. The Reviewer #3 also raised the same concern. Smith (2002) reported with Stick figures(cartoons) that comparing prevalence of bullying cross-nationally is difficult because cultural diversity in the conceptualization and understanding of bullying. We removed it to avoid potential misunderstanding.

Reference:

[1] Smith PK, Cowie H, Olafsson RF, Liefooghe AP, Almeida A, Araki H, del Barrio C, Costabile A, Dekleva B, Houndoumadi A, Kim K, Olafsson RP, Ortega R, Pain J, Pateraki L, Schafer M, Singer M, Smorti A, Toda Y, Tomasson H, Wenxin Z (2002). Definitions of bullying: a comparison of terms used, and age and gender differences, in a fourteen-country international comparison. Child Dev, 73(4):1119-33.

[2] Lotfi S, Dolatshahi B, Mohammadkhani P, Campbell M, Rezaei Dogaheh E (2014). Prevalence of Bullying and its Relationship with Trauma Symptoms in Young Iranian Students. PCP, 2 (4) :247-254

19. Discussion: Line 400: Please replace “improve” with “increase” or similar.

Response: Thanks for pointing it out. We replaced “improve” with “increase” as you suggested.

20. Page 20: Please remove the Data Availability, Funding, Competing Interests sections from the main text, and ensure all information is accurately entered in the Data Availability, Financial Disclosure, and Competing Interests sections of the manuscript submission metadata.

Response: Thanks for pointing it out. We removed the Data Availability, Funding, Competing Interests sections from the main text, and checked the manuscript submission metadata.

21. References: Please use the "Vancouver" style for reference formatting, and see our website for other reference guidelines <https://journals.plos.org/plosmedicine/s/submission-guidelines#loc-references>

Please check the abbreviations for Journal titles: For example, reference 36 should be Lancet Child Adolesc Health.

Response: We used PLOS EndNote style (https://endnote.com/style_download/plos-public-library-of-science-all-journals/) to format our references, and checked the abbreviations for Journal titles.

22. Figure 1: Please clarify whether Japan, Malaysia and Norway did not measure/report data on grade repetition, or whether grade repetition does not occur in these countries (Methods: Line 154-155: “All participants in Japan, Malaysia, and Norway were excluded because no students in the three countries have ever repeated a grade (n=18,033).”

Response: As mentioned in responses to #9, grade repetition does not occur in these three countries. We also revised the Figure 1 accordingly.

23. Table 2 and Table 3: Please do include the versions of the table, with 95% CIs and p values reported for each comparison for bullying victimization and retention between boys and girls.

Response: We added 95% CIs and p values reported for each comparison for bullying victimization and retention between boys and girls in Table 2, and for each comparison for bullying victimization between retained and promoted students in Table 3.

24. Table 4: Please also include the results from the unadjusted models. Please provide the 95% CIs as upper and lower bounds, as well as the p values for each comparison.

Response: The unadjusted OR with 95% CIs and p values for each comparison has been provided in Table 3.

25. S3 Table: Please remove all trademark/copyright symbols from the table (e.g. ,)

Response: We removed all trademark/copyright symbols from the S3 Table.

26. S4 Table, S7 Table, and S10 Table: Please include the results of the unadjusted analyses as well.

Response: We have included the results of the unadjusted analyses in S4 Table, S7 Table, and S10 Table as you suggested.

**Comments from the reviewers:**
Reviewer #1: The authors have responded to each comment in turn, substantially rewording the manuscript where required.

Response: Thanks for your positive comments.

Reviewer #3: The manuscript, Grade repetition and bullying victimization in teenagers: A global cross-sectional study, examines the association between a history of grade retention and experiences of bullying victimization in the past year for adolescents with an average age of about 15.8 years in 74 countries around the world. The study addresses a relatively neglected area of grade retention research by examining its association with bullying victimization. Overall, the paper is stronger but still has some areas that could be improved with further work including strengthening the theoretical rationale and clarifying the measurement and bullying victimization composites. The paper would still benefit from additional significant copy editing due to numerous typos and errors in word selection and grammar.

Response: Thanks for pointing this out. As the authors are all non-native English speakers, we invited Prof. Frank J. Elgar at McGill University, whose research interests also include bullying, to edit our manuscript as you suggested. We acknowledged his effort in the Acknowledgments section. The language issues, if any, are ours.

Authors mention that several theoretical orientations have been proposed to explain why retained students are at higher risk of being bullied but doesn't really describe a theory - authors could go more in depth in detailing a theory that drives their selection of variables. What is missing for me is thought put into how cultural differences between countries may impact the association. For example, authors mention that the degree of stigma of repeating a grade varies by culture - can the author provide some key examples? The theory should be connected to the selection of variables and the interpretation of results. I want to know why authors feel there was an association in some but not all countries. Are there cultural or policy or prevalence differences?

Response: Thanks for your suggestion. In the first round of revision, we focused on specific risk factors. In the revised manuscript, we introduced the social-ecological framework to understanding bullying behaviors, at different levels, including individual, family, school and societal contexts. The framework can help us selecting covariate variables and interpretating the findings.

There is no specific study to our knowledge to quality how the stigma of repeating a grade varies by culture, however, we still can provide some key examples. A study in U.S. revealed that grade retention was the single greatest fear in students, higher than loss of a parent or going blind [1]. Another study in France found that students themselves consider the repeating of a school year as a beneficial policy [2].

It is a very interesting but complicated question on the cultural or policy or prevalence differences. First, it is a very important level in the social-ecological framework of bullying. Religious beliefs, culture and policies are the concrete of societal contexts. Religious beliefs exert a significant amount of influence in youth behaviors and interpersonal relationships. Research shows that higher levels of religious participation were associated with less frequently externalizing problem Behaviors (including bully others) and internalizing problem behaviors [3]. Policy represents another societal level and is important for school bullying. A study conducted in 25 U.S. States suggested that antibullying policies may represent effective intervention strategies for reducing risk of being bullied traditionally or electronically [4].

Second, cultural or policy on bullying varies across country, even within countries. Take antibullying policy for example, Mainland China has not launched its first national law on antibullying until this year (June 1, 2021). In U.S., each jurisdiction, including all 50 states, the District of Columbia and U.S. territories (state), addresses bullying differently [5].

Third, the legislation on antibullying is not effective unless collaborative efforts of homes, schools, and communities. A study conducted in South Korean suggested that, at the school level, enforcement of an anti-bullying policy and/or an anti-bullying program, were not associated with the differences in teacher responses (Ignore and Action) in a bullying incident [6].

The societal contexts (cultural or policy) might be different in different countries. However, we did not collect the indicators as we mentioned in the last part of the Discussion. Studies on bullying in different countries should acknowledged the importance of societal level differences on bullying.

Reference:

[1] Gabrielle E. Anderson, Shane R. Jimerson & Angela D. Whipple (2005) Student Ratings of Stressful Experiences at Home and School, Journal of Applied School Psychology, 21:1, 1-20

[2] Grade repetition. https://www.cnesco.fr/en/grade-repetition-2/

[3] Petts, R. (2009). Fathers' Religious Involvement and Early Childhood Behavior. https://www.semanticscholar.org/paper/Fathers'-Religious-Involvement-and-Early-Childhood-Petts/f26ccc75045a10925110e244f6f2efcbc7289873

[4] Hatzenbuehler ML, Schwab-Reese L, Ranapurwala SI, Hertz MF, Ramirez MR(2015). Associations Between Antibullying Policies and Bullying in 25 States. JAMA Pediatr., 169(10):e152411.

[5] Laws, Policies & Regulations. https://www.stopbullying.gov/resources/laws

[6] Yoon, J., Bauman, S., Choi, T., & Hutchinson, A. S. (2011). How South Korean teachers handle an incident of school bullying. School Psychology International, 32(3), 312–329.

- Authors describe two previous studies - one in Brazil and one in the U.S. - how did the results differ from the Brazil and U.S. results in this study?

Response: As we discussed in Discussion section, the two previous studies with small sample have produced inconsistent results, our study provided a definitive answer at the global level to a debated research question.

- Are higher retention rates related to less association between retention and victimization?

Response: No, we did not observe the relation. For example, as shown in the supplementary tables, the association strength (AOR) is 1.20(1.02-1.40) in Macao where the retention rate is 29.98%, while in Malta the AOR is 1.84(1.26-2.69) and the retention rate is only 4.83%.

- I wonder if the limitation of the sample only being students attending school differs by country as well. Are there differences in school attendance rates for 15year-olds around the globe?

Response: It’s a good question. But for all we know, there is no global data on school attendance rates for 15-year-olds. According to latest UNICEF Global database on adjusted net attendance rate [7], the adjusted net attendance rates in children vary widely across the countries.

Reference:

[7] Adjusted net attendance rate – Percentage of children of a given age that are attending an education level compatible with their age or attending a higher education level. Data source: https://data.unicef.org/wp-content/uploads/2021/04/Adjusted-net-attendance-rate_2021-1.xlsx

Regarding the measurement of victimization, I am a bit unclear on why all three types of measures are needed. Looking at each type and any type of victimization makes sense. How does the composite mean score add value? Would this be a "diversity" of bullying victimization experience? It seems hard to validate such a composite b/c someone could be physically attacked every day but never experienced any of the other types of victimization and they would have a lower score than someone who has been teased a few times a month and excluded a few times a month. The terms "victimization prevalence" and "victimization score" don't help with understanding the differences. I would appreciate a rationale provided for each measure along with a citation with validity evidence to support using this measure from extant literature.

Response: We originally only calculated the prevalence at each type and any type of victimization as you mentioned. In the first round of revision, Reviewer #2 suggested “You also improve measurement precision by creating a composite score as suggested by Vaillancourt et al., 2010. ”

Hence, we added a derived composite score as Reviewer #2 suggested [8,9]. We also cited two published papers on creating a composite score from Prof. Tracy Vaillancourt in University of Ottawa. The victimization score reflects the levels (number of types) of bullying victimization, while the victimization prevalence reflects the percentage of being bullied in specific type or any type.

Reference:

[8] Tracy Vaillancourt, Vi Trinh, Patricia McDougall, Eric Duku, Lesley Cunningham, Charles Cunningham, Shelley Hymel & Kathy Short (2010) Optimizing Population Screening of Bullying in School-Aged Children, Journal of School Violence, 9:3, 233-250

[9] Wang, W., Brittain, H., McDougall, P., & Vaillancourt, T. (2016). Bullying and school transition: Context or development? Child Abuse & Neglect, 51, 237–248.

When describing the participants, it doesn't seem helpful to write that half the participants are lower than the average age (delete) since that is self-evident.

Response: Thanks for pointing this out. We removed the sentence (half the participants are lower than the average age) as you suggested.

In their interpretation of results, authors should discuss the possibility that bullying victimization caused the grade repetition, unless their theoretical orientation or existing empirical literature strongly support a one directional relation.

Response: Grade repetition could be of reciprocal causation with bullying victimization. In the limitation section, we discussed the other directional relation between bullying victimization and grade repetition, and cited two existing empirical literature as you suggested.

There were too many types and wording errors to document but here are a couple of them:

- What is "deprived victimization score"? Perhaps authors mean, "derived"

Response: Thanks for pointing this out. It should be "derived victimization score".

- In third author summary bullet point, I recommend changing "and vary" for "yet prevalence varies"

Response: We revised the bullet point as you suggested.

- I don't understand the sentence on page 18 lines 383-385 "Second, the cultural differences in the bullying definition also partly responsible for the variation because the actual cross-national comparable data on bullying can only be collected by pictures."

Response: Thanks for pointing this out. A possible explanation for the finding (variational prevalence) in the PISA participating countries may reflect the cultural differences in perceptions of bullying behaviors [10].

Smith (2002) used cartoons to examine the comparability of various terms cognate with bullying in different countries and language groups:

Stick figures were used so as to avoid issues of clothing, which might vary by culture, and to avoid suggesting any particular ethnic group or skin color. Each cartoon had a caption in the native language, as listed in Figure 1 below for the English language version [11].

The PISA team developed the bullying scale (eight items) in PISA 2015, and dropped the first two items (“I got called names by other students” and “I got picked on by other students”) based on the exploratory analysis the PISA 2015 data. The efforts were made to produce a scale with comparability across PISA participating countries.

Even though, the interpretation should be examined cautiously as the observed large difference in prevalence might be due to cross-cultural differences or it may be methodological because of not using pictures in assessing bullying [12].

We removed “because the actual cross-national comparable data on bullying can only be collected by pictures” to reduce potential misunderstandings.

Reference:

[10] Chen YY, Elklit A (2017). Exposure to Bullying Among Adolescents Across Nine Countries. J Child Adolesc Trauma. 11(1):121-127.

[11] Smith PK, Cowie H, Olafsson RF, Liefooghe AP(2002). Definitions of bullying: a comparison of terms used, and age and gender differences, in a fourteen–country international comparison. Child Development,73:1119–1133

[12] Lotfi S, Dolatshahi B, Mohammadkhani P, Campbell M, Rezaei Dogaheh E(2014). Prevalence of Bullying and its Relationship with Trauma Symptoms in Young Iranian Students. PCP.; 2 (4) :247-254

Additional minor wording recommendations:

- Delete, "as we mentioned in the background section."

Response: We removed "as we mentioned in the background section" as you suggested.
