# Supplementary material for: Grade repetition and bullying victimization in adolescents: A global cross-sectional study of the Program for International Student Assessment (PISA) data from 2018
Source: PLoS Med. 2021 Nov 11;18(11):e1003846. doi: 10.1371/journal.pmed.1003846 (PMC8584722; doi:10.1371/journal.pmed.1003846)
Supplement: Attachment — Submitted filename: Rebuttal letter3.docx [file pmed.1003846.s011.docx]

Dear Dr. Caitlin Moyer,

We are pleased to submit the revised version of the manuscript titled “Grade repetition and bullying victimization in teenagers: A global cross-sectional study” (PMEDICINE-D-21-00390R4) for consideration in PLOS Medicine’s Special Issue: Global Child Health: From Birth to Adolescence and Beyond.

We thank the Senior and Reviewing Editors of PLOS Medicine, the Special Issue Guest Editors, and external Reviewers for the detailed and constructive comments. We worked on addressing the requests and comments raised by the editors and reviewer #3. We removed the bullying composite score after careful consideration.

We thank the Editors and Reviewer #3 for all their requests and comments, which allowed us to clarify several points and improve our manuscript. And you will find below a point-by-point response to the concerns raised by the editors and reviewer #3.

Please don't hesitate to contact us if you have further questions.

Sincerely yours,

Dr. Qiru Su and Dr. Xiayun Zuo

**Requests from the editors:**

1. From the Academic Editor: Please cite the source of the bullying composite score, indicate whether or not the score has been validated in other study populations, the alpha level of reliability of the composite score in this study population to ensure the composite score is reflective of a single, underlying bullying construct. This information is necessary to support that the scale is sufficiently reliable to be used as a single composite.

Response: Thanks for your comments. Reviewer #3 also raised same concerns. The evidence on validity of the composite score is scarce, however, the practice of the composite score is unusual in the existing body of literature on bullying. We decided to remove the bullying composite score after quite a bit of deliberation, you can view detailed information behind our decision in our response to Reviewer #3.

2. Abstract: Methods and Findings Line 26-27: Please revise to: “...the limited evidence of associations between grade repetition on school bullying is inconsistent…”

Response: We revised this sentence to “the limited evidence of associations between grade repetition and school bullying is inconsistent and ambiguous” as you suggested to avoid causal language.

3. Abstract: Line 51: Please revise to “...having possessions taken away…” or similar.

Response: We revised “…possessions were taken away…” to “...having possessions taken away…” in Abstract and throughout the manuscript as you suggested.

4. Abstract: Conclusions: We suggest opening the first sentence with “In this study, we observed that globally, both boys and girls who ever repeat…”

Response: We revised the first sentence in Conclusion as you suggested.

5. Author summary: Please format these as bullet points.

Response: We revised the Author summary, tried our best to make it objective, brief, succinct, specific and accurate. We hope they meet the requirements.

6. Author summary: Line 79: Please revise to “Grade repetition was associated with bullying victimization in both boys and girls.”

Response: We revised the sentence as you suggested.

7. Author summary: Line 87: Please revise to: “Girls who repeat a grade may be at higher risk of experiencing bullying than boys, and may benefit from targeted interventions.” or similar.

Response: We revised the sentence as you suggested.

8. Introduction: Line 105: Please reference this statement: “...a common misperception is that repeating a grade allows the student to grow academically and socially.”

Response: We added a reference to the statement as you suggested.

9. Introduction: Line 144: This is the first instance where sex differences are mentioned. It would be helpful to have some rationale for your hypothesis that the association between bullying and repetition would be stronger among girls.

Response: We added the rationale and reference for the hypothesis as you suggested.

10. Methods: Line 166: Please cite the PISA website as a reference.

Response: We cited the PISA website as a reference as you suggested.

11. Methods: Please provide more detail here or in the supporting information files (such as S3 Table) describing how the ESCS and parental emotional support scores were calculated from the survey responses.

Response: ESCS and parental emotional support score are the deprived variables that generated by the PISA team at the OECD. The details are complicated and quite long, making it difficult to briefly describe them in our manuscript. We cited the PISA Technical Report for the readers who are interested in the behind process.

12. Methods: Line 210: Please address the comments of the Academic Editor and Reviewer 3. We require that you please provide necessary information to support the use of the composite victimization score, including information pertaining to validity and rationale for its use, and please discuss the limitations of the score.

Response: We removed the composite victimization score in our manuscript, you can find more details in our response to Reviewer #3.

13. Results: Line 310- : Please provide the p values in addition to the 95% CIs for the adjusted ORs for the associations of each type of bullying with school promotion.

Response: We provided the crude ORs (95% CIs) and p values for the associations in this paragraph (Table 3) as you suggested.

14. Results: Line 333-334: Please clarify this sentence: “Across all countries, retained students reported a higher level and higher prevalence of victimization than promoted students.” or similar, depending on your meaning.

Response: We removed “a higher level” which indicating higher composite victimization score, given that we removed the composite score throughout the manuscript.

15. Conclusions: Line 452-453: Please avoid the causal language in the final sentence, and please revise to: “Grade repetition was associated with increased likelihood of bullying victimization…”

Response: We removed the causal language in the final sentence as you suggested.

16. References: Please check that the citation for reference 1 is complete.

Response: We updated the citation for reference 1. The reference is available from the page https://op.europa.eu/en/publication-detail/-/publication/10f14860-12a3-4f9e-b10a-32bee875420d

17. S8 Table: Please remove the extra parentheses at the top of the “Adjusted” column under Odds ratio.

Response: Thanks for point it out. We removed the extra parentheses in the table as you suggested.

**Comments from the reviewers:**
Reviewer #3: Overall the paper is stronger in this revision, however, the authors did not adequately address my concerns about the measurement of victimization. The construct of bullying cannot be measured by creating a sum score of frequency responses to a diversity of types of victimization experiences and using it as a continuous item. I do not believe a reviewer was justified in recommending this practice, it does not make conceptual sense as bullying victimization experiences (e.g., verbal, physical, relational, cyber) do not all need to occur for someone to be severely bullied. The authors cite Vaillancourt et al. (2010) as recommending this process but, from my reading, their research recommended using a measure of behavioral based bullying with a cut-off score for a binary determination of yes/no bullied with no=never and yes=any bullying. Thus, if the authors and editor feel that the reviewer feedback is important to comply with and that a composite measure of bullying victimization is critical to answering their research questions, they should provide adequate explanation for and documentation of why this approach is valid and what this approach is measuring. I recommend Green et al. (2013) and a couple of additional articles about measuring bullying in informing the authors' decision.

Green, J. G., Felix, E. D., Sharkey, J. D., Furlong, M. J., & Kras, J. E. (2013). Identifying bully victims: Definitional versus behavioral approaches. Psychological Assessment, 25, 651-657.

Sharkey, J. D., Ruderman, M. A., Mayworm, A. M., Green, J. G., Furlong, M. J., Rivera, N., & Purisch, L. (2015). Psychosocial functioning of bullied youth who adopt versus deny the bullied victim label. School Psychology Quarterly, 30, 91-104.

Felix, E. D., Sharkey, J. D., Green, J. G., Furlong, M., F., & Tanigawa, D. (2011). Getting precise and pragmatic about the assessment of bullying: The development of the California Bullying Victimization Scale. Aggressive Behavior, 37, 234-247.

The rest of my feedback was adequately addressed.

Response: Thanks for your comments and recommended articles. We decided to remove the composite score from our manuscript after internal discussions and much thought, given the interpretation of the composite score is a little ambiguous, the evidence on validity of the composite score is scarce and the composite score is not critical to answering our research questions.

Although we removed the composite score from our manuscript, we politely disagree on your statement that: “The construct of bullying cannot be measured by creating a sum score of frequency responses to a diversity of types of victimization experiences and using it as a continuous item”. Theoretically, composite score has greater sensitivity and variability than a binary outcome (all-or-nothing experience) and reflects the continuous nature of the latent victimization variable. Although the evidence regarding the construct validity of scores from multi-item scales to measure the continuous latent constructs of bullying victimization is relatively little, there are many practices in the literatures (listed at the end of the letter).

We agree on your statement that: “they should provide adequate explanation for and documentation of why this approach is valid and what this approach is measuring”. Even this approach is not unusual, the evidence on its validity is scarce and composite score is intuitively non-interpretable.

Reference:

The Forms of Bullying Scale (FBS): Validity and reliability estimates for a measure of bullying victimization and perpetration in adolescence

<https://ro.ecu.edu.au/cgi/viewcontent.cgi?referer=&httpsredir=1&article=1743&context=ecuworks2013>

Development of a Measure of the Experience of Being Bullied in Youth

https://pubmed.ncbi.nlm.nih.gov/22250594/

In the previous version of our manuscript, we cited two articles from Vaillancourt. In the part “to improve measurement precision” we cited Vaillancourt et al. (2010) the article you read. In the part how we generated the score, we cited his recently published article (2016), a composite victimization score was created for each participant by taking the average of all five items:

“Participants were then asked to respond to a series of five questions about their experiences with bullying, “Since the start of the school year (September). . .How often have you been bullied at school?” using a 5-point frequency scale (0 = Not at all; 1 = Only a few times this year; 2 = Every month; 3 = Every week; 4 = Many times a week). Participants first responded to this general peer victimization question and then responded to four questions about different forms of peer victimization (physical, verbal, social, and cyber victimization). A composite victimization score was created for each participant by taking the average of all five items with higher scores indicating higher levels of peer victimization. The peer victimization measure had good reliability at both time points (T1 ˛= 0.80; T2 ˛= 0.81).”

Source: Bullying and school transition: Context or development?

<https://www.sciencedirect.com/science/article/abs/pii/S0145213415003622>

In addition to that, we also find other articles that using the composite score, for example:

1. “The Bullying Subscale contained 6 items that measured the frequency of harassing, upsetting, or making fun of other students; spreading rumors; starting arguments; or socially

excluding others (0 = never, 1 = 1-2 times, 2 = 3-4 times, 3 = ≥5 times; α = 0.87). The Victimization Subscale contained 4 items that measured the frequency of being picked on, made fun of, called names, and hit or pushed by other students (0 = never, 1 = 1-2 times, 2 = 3-4 times, 3 = ≥5 times; α = 0.86). Bullying perpetration scores ranged from 0 to 18 and victimization scores ranged from 0 to 12.”

Source: Cyberbullying Victimization and Mental Health in Adolescents and the Moderating Role of Family Dinners

https://jamanetwork.com/journals/jamapediatrics/fullarticle/1900477

2.“Overall, 11 types of bullying were presented in the questionnaire: five concerned direct bullying, either verbal or physical (teasing about physical appearance; teasing for other reasons; verbal offenses; physical aggression, e.g. being hit or kicked; being threatened), six concerned indirect or relational bullying (ignored by peers; stealing money or other things; spreading rumors; exclusion from sports activities; exclusion from other group activities; exclusion from parties). …… The participants were asked to fill out the questions: “how often have you been bullied at school in the past couple of months?”; “how often have you taken part in bullying another student(s) at school in the past couple of months?” using a five point scale (“never involved”, “only once or twice”, “2 or 3 times a month”, “about once a week”, “several times a week”). ……To code involvement in bullying or victimization, both categorical and dimensional approaches were used. In the categorical approach, individuals were classified as bullies, victims and bully-victims if they exceeded a cut-off of two or three episodes a month or more (respectively as bully, victim or both) in an established period (the last two months), according to standard procedures [34]. So, occasional involvement as a bully and/or victim was not considered. In the dimensional approach, a continuous score of single bullying behavior or victimization was considered by assigning point values to each item from 1 (never involved) to 5 (several times a week). In addition, a total score of bullying or victimization was computed by summing the scores of all items of bullying and victimization respectively. Higher scores indicate a greater frequency of engaging in bullying behavior or victimization.”

Source: Bullying and Victimization in Overweight and Obese Outpatient Children and Adolescents: An Italian Multicentric Study https://journals.plos.org/plosone/article?id=10.1371/journal.pone.0142715

3.“The primary outcome measure consisted of 12 items from the Revised Olweus Bully/Victim Questionnaire (Olweus, 1996) that asked students to report the frequency at which they bullied others (e.g., “I called students mean names, made fun of, or teased”) and experienced bully

victimization (e.g., “I was called mean names, made fun of, or teased”) on a 5-point scale ranging from 1 (It hasn’t happened since the beginning of the school year) to 5 (Several times a week). Program effects were found on rates of victimization over the course of the study, with the strongest effects found in Grades 4 and 6 ( Jenson et al., 2013; Jenson & Dieterich, 2007).

For this study, we focus on bully-victimization scores in the spring semester of 5th grade at the conclusion of YM implementation. Six bully-victimization items were averaged to create a single composite score and rescaled to range from 0–4 (M 5 0.72, SD 5 0.82, skewness 5 1.72, kurtosis 5 2.79). Scores of zero, therefore, better reflect the response option (It hasn’t happened) for the lowest category of the scale.”

Source: Analyzing Skewed Continuous Outcomes With Many Zeros: A Tutorial for Social Work and Youth Prevention Science Researchers

<https://www.journals.uchicago.edu/doi/pdf/10.1086/701235>

4. [ Items 3, 11,27, are reverse scored so that each item is scored from 1 (really true for me) to 4 (really true for me), higher scores indicating greater experience of victimisation. For each scale, the total score is computed according to Harter (1985) (i.e., sum of 6 items/6) so that each total scale score can range from 1 to 4]

Source: Assessment of bully/victim problems in 8 to 11 year-olds

<https://www.researchgate.net/publication/260744526_Austin_Joseph>

5. The Social Acceptance and Bullying Dimension includes three questions: “Have you been afraid of other girls and boys?”, “Have other girls and boys made fun of you?”, and “Have other girls and boys bullied you?”. Responses are made on a 5-point Likert scale [never (0), seldom (1), quite often (2), often (3), and always (4)]. An item level mean bullying score is computed from the three questions with higher scores indicating more bullying.

Source: BULLYING OF EXTREMELY LOW BIRTH WEIGHT CHILDREN: ASSOCIATED RISK FACTORS DURING ADOLESCENCE

https://www.ncbi.nlm.nih.gov/pmc/articles/PMC3625459/

6. To measure the traditional bullying perpetration, the Italian version of the bully subscale of the Illinois Bully Scale (IBS-B) [39] was administered. The Illinois Bully Scale [84] is a self-reported scale that includes 18 items divided into 3 subscales: bully (9-item; e.g., “I annoyed other students”), victim (4-item; e.g., “Other students beat and pushed me”), and fighting (5-item; e.g., “If someone beats me firstly, I will beat him/her”). In this study, for each item of the IBS-B, participants were asked to indicate the frequencies with which they carried out the described behavior. Responses were collected on a 5-point scale, which considered the following alternatives: “never” = 0, “1 or 2 times” = 1, “3 or 4 times” = 2, “5 or 6 times” = 3, “7 or more times” = 4. The good psychometric properties of the Italian IBS-B have been described in Catone et al. [39]. As indicated by Espelage and Holt [84] a total score of traditional bullying perpetration was computed for each participant (ranges: 0 to 36), with higher scores indicating higher self-reported bullying behaviors. Cronbach’s Alpha = 0.824; ωt = 0.859.

To measure the cyberbullying perpetration [[**40**](https://www.mdpi.com/2076-3425/11/8/1059/htm#B40-brainsci-11-01059)], the Italian version of the Smith’s cyberbullying scale (SCBS) was administered. The SCBS [[**56**](https://www.mdpi.com/2076-3425/11/8/1059/htm#B56-brainsci-11-01059)] is a self-report scale assessing cyberbullying behaviors by considering seven different media (7-item): text messaging; pictures/photos or video clips; phone calls; email; chat rooms; instant messaging; and websites. Participants were preliminarily presented a definition of cyberbullying behaviors then were asked to indicate for each media if they bullied others through it during the last year. Responses were collected on a 5-points scale that considered the following alternatives: “never” = 0; “only once or twice” = 1; “two or three times a month” = 2; “about once a week” = 3; “several times a week” = 4). Although the Italian adaptation of the scale has been already used, its psychometric characteristics have not been described. For this reason, the dimensionality and reliability of the SCBS were preliminarily verified before running the main analyses of this study. A confirmatory factorial analysis carried out on the total sample confirmed the unidimensional structure of the scale, χ^2^(14) = 205.31, *p* < 0.001, *RMSEA* = 0.068, 90% *CI* [0.060; 0.076], *CFI* = 0.976, *SRMR* = 0.026, *N* = 2959; whereas the reliability analysis showed an adequate value, Cronbach’s Alpha = 0.795; ω_t_ = 0.889. Therefore, as indicated by Smith and colleagues [[**56**](https://www.mdpi.com/2076-3425/11/8/1059/htm#B56-brainsci-11-01059)], a total score of cyberbullying perpetration was computed for each participant (ranges: 0 to 28), with higher scores indicating higher self-reported cyberbullying behaviors.

Source: The Relation of Callous–Unemotional Traits and Bullying in Early Adolescence Is Independent from Sex and Age and Moderated by Conduct Problems

https://www.mdpi.com/2076-3425/11/8/1059/htm

7. “Bullying and victimization were measured by four items each, scored on a five-point scale: from 0 (never), 1 (once or twice), 2 (two or three times a month), 3 (about once a week), and 4 (several times a week). The items for victimization assessed (1) ‘how often has the child been victimized in the past couple of months? (in general)’, (2) ‘how often has the child been teased, laughed at, or called names in the past couple of months? (verbal victimization)’, (3) ‘how often has the child been physically victimized, such as being hit, kicked, and pushed in the past couple of months? (physical victimization)’, and (4) ‘how often has the child been excluded by other children, ignored, or have other students spread false rumors? (relational victimization)’. Bullying was assessed with the same items, but in the perpetration form (see Jansen et al. (2012)). The total score of both phenotypes could range from 0 to 16.”

Source: Bullying and Victimization: The Effect of Close Companionship

https://www.cambridge.org/core/journals/twin-research-and-human-genetics/article/bullying-and-victimization-the-effect-of-close-companionship/38789B15793DA0D2A5019C2FBB14EC8E

8. Childhood bully victimization was assessed with the victimization subscale of the Illinois Bully Scale [33]. The subscale consists of four questions that measure both physical and verbal victimization that individuals experience from or by peers. The responses to each question ranged from never (scored 0) to 1–2 times (1), 3–4 times (2), 5–6 times (3), and 7 or more times (4). The responses were summed to derive a total score that ranged from 0 to 16. The verbal bullying, physical bullying, and bully victimization subscales of the Illinois Bully Scale were validated among 394 pupils in two secondary school in semi-urban southeast Nigeria. The Cronbach’s alphas for the whole scale and the victimization subscale were 0.84 and 0.78, respectively [17].

Source: Internal consistency and correlation of the adverse childhood experiences, bully victimization, self-esteem, resilience, and social support scales in Nigerian children

https://bmcresnotes.biomedcentral.com/articles/10.1186/s13104-020-05174-3

9. California Bullying Victimization Scale (CBVS) [14]: The CBVS is a self-report questionnaire which assesses eight forms of bullying victimization including being teased, spreading of rumors behind back, being ignored deliberately, physically being hurt, threatened, making sexual comments, jokes, or gestures, had things stolen, and teased and threatened through the Internet. Each item is rated on a five-point scale ranging from never to several times a week and assigned scores from 0 to 4. Total victimization score ranges from 0 to 32 with higher scores indicating greater victimization. The CBVS also assesses eight forms of bullying perpetration and these are scored in a similar fashion. The scale also yields a total bullying perpetration score. In the present study, we identified and compared two groups of students: pure victims of bullying (had never bullied others) and students not involved in any form of bullying (neither victims nor bullies).

Source: School Bullying and Association with Somatic Complaints in Victimized Children

https://link.springer.com/article/10.1007/s12098-020-03620-5
