# Supplementary material for: Grade repetition and bullying victimization in adolescents: A global cross-sectional study of the Program for International Student Assessment (PISA) data from 2018
Source: PLoS Med. 2021 Nov 11;18(11):e1003846. doi: 10.1371/journal.pmed.1003846 (PMC8584722; doi:10.1371/journal.pmed.1003846)
Supplement: Attachment — Submitted filename: Rebuttal letter4.docx [file pmed.1003846.s012.docx]

Dear Dr. Caitlin Moyer,

We are pleased to submit the revised version of the manuscript titled “Grade repetition and bullying victimization in adolescents: A global cross-sectional study of the Program for International Student Assessment (PISA) data from 2018” (PMEDICINE-D-21-00390R5) for consideration in PLOS Medicine’s Special Issue: Global Child Health: From Birth to Adolescence and Beyond.

We thank the Editors for all their requests and comments, which allowed us to clarify several points and improve our manuscript. And you will find below a point-by-point response to the concerns raised by the editors.

Please don't hesitate to contact us if you have further questions.

Sincerely yours,

Dr. Qiru Su and Dr. Xiayun Zuo

**Requests from the editors:**

1. From the academic editor: Given that the authors report an alpha reliability for the scale of 0.88, it is unclear why they removed the composite scale. It also is unclear whether the alpha that is reported is from the original article describing the scale or whether it is from the sample under study. If the former, this should be stated, and the alpha for the sample should be provided. If the latter, it is unclear why the authors do not use a composite score, as an alpha of 0.88 is adequate evidence justifying the use of a composite score. In sum, the descriptions of the outcome measure needs to be cleaned up and the justification for using whatever measurement scale for the outcome(s) needs to be more clearly justified.

Response: Thanks for pointing it out. The Cronbach's alpha (0.88) was calculated from the sample under study, we clarified this as you suggested.

We agree with you that an alpha of 0.88 is adequate evidence justifying the use of a composite score. And composite score is also not unusual in published articles on bullying as we mentioned in 3rd rebuttal letter. We introduced the composite score but removed it eventually based on the following considering:

1) Lack of evidence. We did not find the documentation why this approach is valid. Usually, the scale developers will use statistical analyses to devise a recommended scoring methodology for generating composite score, to ensure it being calculated consistently. The PISA team, however, does not develop or recommend any method to developing the composite score of the school bullying. Types of composite measures include 1) All-or-none/any-or-none scoring, 2) Sum, 3) Average, 4) Weighted average, and 5) Opportunity scoring [1]. We used the common average score as the composite score, which has not been validated by in PISA team in the same population or other researchers in other study populations.

2) Choosing whatever measurement scale for the outcome(s) depends on its importance to answering our research questions. We want to highlight that grade repetition is associated each type of bullying victimization, including physical bullying victimization. It’s our key research findings and the composite score of bullying victimization could not do that.

The composite score as a single variable can be used to investigate the association between grade repetition and overall bullying victimization, with higher victimization score indicating higher level of bullying victimization. But not everyone is a fan of this approach, as commented in 3rd round by Reviewer #3: “The construct of bullying cannot be measured by creating a sum score of frequency responses to a diversity of types of victimization experiences and using it as a continuous item.” Furthermore, existing any type of bullying victimization can provide this information partly, although not exact same as victimization score does.

In short, we introduced composite score of bullying victimization in 2rd version of our manuscript and removed the composite score eventually given that the evidence on validity of the composite score is scarce and the composite score is not critical to answering our research questions.

Source:

[1] Karen Pace. Composite Measures.

https://www.healthit.gov/sites/default/files/pdf/2012-08-09-behavioral-health-clinical-quality-measures-technical-expert-panel-composite-measures.pdf

2. Title: We suggest revising the title to “Grade repetition and bullying victimization in adolescents: A global cross-sectional study of the Program for International Student Assessment (PISA) data from 2018.

Response: Thanks for your suggestion, we revised the manuscript title as you suggested.

3. Throughout: If possible, please replace “retained students” or “retained girls” etc. with “students who repeat a grade” or similar.

Response: We replaced “retained students” or “retained girls” or “retained boys” with “students who repeat a grade” or “grade repeaters” where possible throughout the manuscript as you suggested.

Source: Sit, P., Cheung, K., Cheong, W., Mak, S., Soh, K., & Ieong, M. (2015). What studying problems are faced by the adolescent grade repeaters in Macao: uncovering underlying mechanisms based on evidences from the PISA 2012 Study. Asia Pacific Education Review, 16(3), 367–377. doi:10.1007/s12564-015-9388-3

4. Abstract: Lines 24-25: Please clarify what is meant by primary in the sentence “Globally around 32.2 million students repeated a grade in primary in 2010.” Please revise if this should read “in primary school” or “in the primary education level” or similar.

Response: We revised it to “in the primary education level” as you suggested.

5. Abstract: Line 49: Please revise “were associated with” to “retained girls had higher risks of” or similar.

Response: We revised it to “girls who repeat a grade had higher risks of” as you suggested.

6. Abstract: Line 58: Please revise “from repeating a grade than boys” to “...risks of specific types of bullying associated with repeating a grade than boys.”

Response: Thanks for pointing it out. We revised this sentence and throughout the manuscript as you suggested to avoid causal implications.

7. Author summary: Line 84: We suggest revising to “The experience of repeating a grade may suggest a need for bullying interventions among both boys and girls”

Response: We revised this sentence as you suggested.

8. Author summary: Line 86-87: We suggest revising to “Girls who repeat a grade may be at higher risk of experiencing some forms of bullying than boys, and may benefit from targeted interventions.”

Response: We revised this sentence to “Girls who repeat a grade may be at higher risk of experiencing some types of bullying than boys, and may benefit from targeted interventions.” as you suggested.

9. Methods: Lines 198-209: As noted by the academic editor, please clarify whether the alpha here represents the original study, and provide the alpha for the sample if so. Please provide a clear explanation and rationale for the bullying victimization outcome used.

Response: We provided with the Cronbach's alpha, which in our example is 0.88. We clarified this as you suggested. We followed the threshold for classification recommended by Dan Olweus, the one that is usually recommended in bullying research for a number of reasons (see, e.g., Solberg & Olweus, 2003). We provided detailed rationale for outcome measurement in our response to the academic editor.

Source: Solberg ME, Olweus D. Prevalence estimation of school bullying with the Olweus Bully/Victim Questionnaire. Aggressive Behavior. 2003;29(3):239-68. doi: 10.1002/ab.10047.

10. Methods: Lines 215-218, 245-246; 253-254: Reference 33 cited here is listed as “forthcoming” in the reference list. There are a number of details of the methods that reference this technical report, please update the reference in the event the 2018 report is available now, or otherwise clarify how the PISA 2018 technical report may be accessed.

Response: Thanks for pointing it out. All chapters of the PISA 2018 technical report are available now except Chapter 1. The report will be published when all chapters are completed. Before that, the report can be accessed at: <https://www.oecd.org/pisa/data/pisa2018technicalreport/>

We updated the reference and give the link that the report can be accessed at now for readers.

11. Results Lines 314-317: “The sex-specific analyses produced similar results in boys and girls (Table 4). Furthermore, interaction analyses showed that retained girls were at higher risk of being made fun of, being threatened, having possessions taken away, and being pushed around compared with retained boys (S5 Table).” We suggest making it more clear that while sex differences were not apparent overall for bullying victimization, there were interactions indicating that for subtypes of bullying, there were differences between girls and boys who repeated a grade.

Response: Thanks for your suggestion, we rephased this paragraph and made it clearer.

12. Discussion: Line 376: Please remove the word “undoubtedly” from the sentence, and please revise “As predicted” to “Consistent with previous work” or “Consistent with our hypothesis” or similar.

Response: We removed the word “undoubtedly” and revised “As predicted” to “Consistent with our hypothesis” as you suggested.

13. Discussion: Line 394: Please revise “than the promoted low achievers” to “than students who are promoted but who may have lower academic achievement” or similar.

Response: We revised “than the promoted low achievers” to “than promoted students who may have lower academic achievement” as you suggested.

14. Conclusion: Line 444: Please revise to avoid causal implications, we suggest: “girls are more likely than boys to experience specific types of bullying associated with repeating a grade.”

Response: Thanks for pointing it out. We revised this sentence as you suggested to avoid causal implications as we did in Abstract (Line 58) and throughout the manuscript.

**Comments from the reviewers:**
Reviewer #3: I appreciate your careful consideration of the composite of bullying and ultimate removal of the composite from the study.

Response: Thanks for your positive comments.
