# Supplementary material for: Grade repetition and bullying victimization in adolescents: A global cross-sectional study of the Program for International Student Assessment (PISA) data from 2018
Source: PLoS Med. 2021 Nov 11;18(11):e1003846. doi: 10.1371/journal.pmed.1003846 (PMC8584722; doi:10.1371/journal.pmed.1003846)
Supplement: Attachment — Submitted filename: Rebuttal letter5.docx [file pmed.1003846.s013.docx]

Dear Dr. Caitlin Moyer,

We are pleased to submit the revised version of the manuscript titled “Grade repetition and bullying victimization in adolescents: A global cross-sectional study of the Program for International Student Assessment (PISA) data from 2018” (PMEDICINE-D-21-00390R6) for consideration in PLOS Medicine’s Special Issue: Global Child Health: From Birth to Adolescence and Beyond.

We thank the Editors for all their comments and suggestions, and revised our manuscript accordingly. And you will find below a point-by-point response to the concerns raised by the editors.

Please don't hesitate to contact us if you have further questions.

Sincerely yours,

Dr. Qiru Su and Dr. Xiayun Zuo

**Requests from Editors:**

1. Abstract: Conclusions: Line 59-61: Please remove the word “robust” at line 59. We suggest revising the final sentence slightly to: “Sex differences in risk of experiencing some subtypes of bullying suggest that tailored interventions for girls who repeat a grade may be warranted.” or similar, as the study findings do not seem to exactly imply that girls could benefit more than boys from a future intervention.

Response: Thanks for your suggestion, we removed the word “robust” at line 59 and Line 443. We revised the final sentence as you suggested in Abstract section, we also revised the sentence at Line 86 in Author summary accordingly.

2. Methods: Line 207-211: Please clarify the description slightly that for the six individual bullying experiences the response options for 0= never or almost never and 1=a few times a year were combined into 0 (a few times a year or less frequently) and the response options for 2= a few times a month and 3= once a week or more were combined into 1 (a few times a month or more frequently). For the combined measure, please make it clear if “0 (never on all types)” corresponds to the “never or almost never” original response to the question, and 1 (any involvement) corresponds to the combination of responses 1, 2, or 3.

Response: Thanks for pointing it out. We revised the description on the measurement of bullying to eliminate the ambiguity at Line 205-214.

Specifically, for the six individual bullying experiences the response options for 0= never or almost never and 1=a few times a year were combined into 0 (a few times a year or less frequently) and the response options for 2= a few times a month and 3= once a week or more were combined into 1 (a few times a month or more frequently).

Similarly, for the combined measure, 0 (never or rare on all types)” corresponds to the combination of original responses 0 or 1 (0= never or almost never or 1=a few times a year) to the question, and 1 (any involvement) corresponds to the combination of original responses 2 or 3 (2= a few times a month or 3= once a week or more).

3. Methods: Line 225-229: We suggest replacing “immigrant” with “migrant” as this term may imply a more neutral point of view. Please replace immigrant/immigration status throughout, including Table 1.

Response: Thanks for your suggestion, we replaced “immigrant” with “migrant” and replaced “immigrant/immigration status” with “migrant status” throughout the manuscript, including Tables (Table 1 and Table 4) and supporting files (S3 Table, S5 Table, and S8 Table).

4. Results: Line 291: We suggest avoiding the term “immigrant” and revising to “...reported a non-native status” or similar.

Response: Thanks for your suggestion, we replaced “immigrants” with a more neutral term “migrants” as you suggested in #3.

5. Discussion: Line 344-345: We suggest revising slightly to: “Third, girls who repeated a

grade are more likely to report having experienced specific types of bullying than boys.”

Response: Thanks for your suggestion, we revised it to “… report having experienced specific types of bullying than boys” as you suggested.

6. Discussion: Line 363: Please revise this sentence if this should read “...have so far focused on its academic outcomes…”

Response: Thanks for your suggestion, we revised this sentence by adding “on” after “focused” as you suggested.

7. Discussion: Line 400-401: We suggest revising this sentence slightly: “The higher risk of experiencing some types of bullying among girls who repeated a grade suggests the possibility that targeted anti-bullying prevention interventions may be especially beneficial for girls.” or similar.

Response: Thanks for your suggestion, we revised this sentence as you suggested.

8. Discussion: Lines 438-441: We suggest revising the sentence to read: “The observed link raises the possibility that the widespread educational policy of grade repetition may partly contribute to the differences in bullying victimization and adds to the evidence against the policy of grade repetition; however, our study cannot establish a causal relationship.” or similar.

Response: Thanks for your suggestion, we revised this sentence as you suggested. Also, we removed “robust” in the sentence at Line 443, as you suggested in #1.

9. Social Media: To help us extend the reach of your research in the event that your article is accepted for publication, please provide any Twitter handle(s) that would be appropriate to tag, including your own, your coauthors’, your institution, funder, or lab.

Response: Thanks for your suggestion, we are happy to but we can not access the Twitter.com from Chinese Mainland. We will extend the reach of our research using social media, including Weibo and WeChat in China.
